# Gene and Transposable Element Expression Evolution Following Recent and Past Polyploidy Events in *Spartina* (Poaceae)

**DOI:** 10.3389/fgene.2021.589160

**Published:** 2021-03-25

**Authors:** Delphine Giraud, Oscar Lima, Mathieu Rousseau-Gueutin, Armel Salmon, Malika Aïnouche

**Affiliations:** ^1^UMR CNRS 6553 Ecosystèmes, Biodiversité, Evolution (ECOBIO), Université de Rennes 1, Rennes, France; ^2^IGEPP, INRAE, Institut Agro, Univ Rennes, Le Rheu, France

**Keywords:** allopolyploidy, hybridization, transcriptome evolution, transposable elements (TE), *Spartina*

## Abstract

Gene expression dynamics is a key component of polyploid evolution, varying in nature, intensity, and temporal scales, most particularly in allopolyploids, where two or more sub-genomes from differentiated parental species and different repeat contents are merged. Here, we investigated transcriptome evolution at different evolutionary time scales among tetraploid, hexaploid, and neododecaploid *Spartina* species (Poaceae, Chloridoideae) that successively diverged in the last 6–10 my, at the origin of differential phenotypic and ecological traits. Of particular interest are the recent (19th century) hybridizations between the two hexaploids *Spartina alterniflora* (2*n* = 6*x* = 62) and *S. maritima* (2*n* = 6*x* = 60) that resulted in two sterile F1 hybrids: *Spartina* × *townsendii* (2*n* = 6*x* = 62) in England and *Spartina* × *neyrautii* (2*n* = 6*x* = 62) in France. Whole genome duplication of *S.* × *townsendii* gave rise to the invasive neo-allododecaploid species *Spartina anglica* (2*n* = 12*x* = 124). New transcriptome assemblies and annotations for tetraploids and the enrichment of previously published reference transcriptomes for hexaploids and the allododecaploid allowed identifying 42,423 clusters of orthologs and distinguishing 21 transcribed transposable element (TE) lineages across the seven investigated *Spartina* species. In 4*x* and 6*x* mesopolyploids, gene and TE expression changes were consistent with phylogenetic relationships and divergence, revealing weak expression differences in the tetraploid sister species *Spartina bakeri* and *Spartina versicolor* (<2 my divergence time) compared to marked transcriptome divergence between the hexaploids *S. alterniflora* and *S. maritima* that diverged 2–4 mya. Differentially expressed genes were involved in glycolysis, post-transcriptional protein modifications, epidermis development, biosynthesis of carotenoids. Most detected TE lineages (except *SINE* elements) were found more expressed in hexaploids than in tetraploids, in line with their abundance in the corresponding genomes. Comparatively, an astonishing (52%) expression repatterning and deviation from parental additivity were observed following recent reticulate evolution (involving the F1 hybrids and the neo-allododecaploid *S. anglica*), with various patterns of biased homoeologous gene expression, including genes involved in epigenetic regulation. Downregulation of TEs was observed in both hybrids and accentuated in the neo-allopolyploid. Our results reinforce the view that allopolyploidy represents springboards to new regulatory patterns, offering to worldwide invasive species, such as *S. anglica*, the opportunity to colonize stressful and fluctuating environments on saltmarshes.

## Introduction

Gene expression dynamics is a key component of polyploid evolution, and has received considerable interest in the last decades, where studies on various polyploid systems revealed an important role of whole genome duplication (WGD) in modulating diverse and novel gene expression patterns (Chen, 2007; Flagel et al., 2008; Yoo et al., 2014). Comparative analyses indicate that the evolution of duplicated gene expression has a temporal dimension, and that immediate responses to polyploidy may not only last over long periods, but also contribute to the long-term processes of diploidization and fractionation (Wendel et al., 2018). One of the key parameters of this dynamics is the divergence level between the genomes being merged and duplicated during the polyploid speciation process (Buggs et al., 2012; Tayalé and Parisod, 2013). Neopolyploids are usually classified as autopolyploids, where homologous genomes (i.e., within species) are duplicated, or as allopolyploids involving duplication of more or less divergent (homoelogous) genomes reunited in the same nucleus following interspecific hybridization (Stebbins, 1971; reviewed in Doyle et al., 2008). Most autopolyploids so far explored exhibit moderate transcriptome or proteome alteration compared to their diploid progenitors (Albertin et al., 2005; Parisod et al., 2010b; del Pozo and Ramirez-Parra, 2014; Visger et al., 2019; Song et al., 2020). In contrast, allopolyploidy seems to be accompanied by profound parental expression repatterning in naturally formed neopolyploids (Hegarty et al., 2006; Chelaifa et al., 2010b; Buggs et al., 2011), experimentally resynthesized allopolyploids and/or their naturally established counterparts (Wendel, 2000; Chen and Ni, 2006; Ha et al., 2009; Soltis et al., 2015), such as in oilseed rape, wheat, cotton, or coffea (Akhunova et al., 2010; Higgins et al., 2012; Combes et al., 2013; Yoo et al., 2013; Wu et al., 2018). These dynamics reflect both the effects of the reunion of divergent regulatory networks (resulting from hybridization) and the effects of genetic redundancy (resulting from WGD), all these contributing to a “transcriptomic shock” (Hegarty et al., 2006), as a functional extension of what was earlier termed as “genomic shock” (McClintock, 1984), including its associated genetic and epigenetic regulatory processes (Hu and Wendel, 2019).

Duplicated genes may undergo partitioning of ancestral functions (subfunctionalization, Force et al., 1999) or benefit from mutations conferring new functionality (neofunctionalization, Ohno, 1970), which in turn affects the long-term retention of the duplicated copies (duplication-degenerescence-complementation model, Lynch and Force, 2000). The enhanced functional plasticity resulting from gene copy redundancy and allelic diversity facilitates the emergence of novel variation, traits and phenotypes (Finigan et al., 2012), thus contributing to species adaptation and long-term diversification (Comai, 2005; Conant and Wolfe, 2008; Jackson and Chen, 2010; Madlung, 2013; Tank et al., 2015; Van de Peer et al., 2017).

Expression evolution in polyploids has been extensively explored by testing their deviation from an expected transcriptomic “parental additivity.” This non-additive parental expression may be perceived by considering either the overall gene expression level (usually measured by comparisons with the average expression of both parental species, i.e., Mid-Parent Value; MPV) or by considering the relative contribution of each homoeologous copy to the total expression level (Grover et al., 2012). The high frequency of biases in the respective contributions of homeologs reported in various allopolyploids lead to the “genomic dominance” concept whereby one parental (homoeologous) subgenome expression state is exhibited in strong preference over the other parental expression state (Flagel and Wendel, 2009).

Recent studies provide accumulating evidence that the “dominant” subgenome is early established following hybridization and may be maintained over generations (Edger et al., 2017; Bird et al., 2018). This, very interestingly, permits to connect the short-term genome dominance phenomenon to the long-term fractionation process affecting polyploid genomes (Wendel et al., 2018), where biases in gene loss between duplicated homoeologous genomes result from the selection against loss of the most expressed copy (Schnable et al., 2011). The corollary is that following allopolyploidy, the “dominant” subgenome (the highest expressed) is more likely to be retained than the “recessive” subgenome (the lowest expressed) that becomes the most fractionated (Cheng et al., 2018). As expected, subgenome dominance is not observed in neo-autopolyploids (Garsmeur et al., 2014) suggesting a major role of the composition of the two subgenomes being merged in this process, notably in their content of transposable or regulatory elements.

Allopolyploidy merges and duplicates more or less differentiated genomes, including repetitive sequences that represent a dynamic component of plant genomes (Bennetzen and Wang, 2014). Transposable elements (TEs) affect in various ways the structure and expression of polyploid genomes. Several studies (reviewed in Vicient and Casacuberta, 2017) have reported transcriptional reactivation of some TE lineages in hybrids and allopolyploids or new insertions as found in tobacco, sunflower, wheat, or *Brachiaria* sp. (Ungerer et al., 2006; Petit et al., 2010; Yaakov and Kashkush, 2012; Santos et al., 2015). As TE expression is controlled by epigenetic regulation (e.g., DNA hypermethylation and siRNAs), new TE regulation following polyploid speciation may result in altered neighbor gene expression including gene silencing and novel gene expression patterns (Kashkush et al., 2003; Hollister and Gaut, 2009; Parisod et al., 2010a; Zhang et al., 2015; Lerat et al., 2019). In allopolyploids, divergence between the parental genomes in terms of TE abundance and distribution is expected to increase subgenome dominance, as the TE-rich subgenome is expected to be targeted by epigenetic regulation and then less expressed than the TE-sparse subgenome (Bird et al., 2018; Bottani et al., 2018).

This complex interplay between parental genome legacy, the challenges faced by the needed coordination of divergent regulatory networks in the same nucleus and the connected processes taking place at the early and latest stages of polyploid species evolution raise critical questions about the rules and mechanisms shaping modern extant genomes and their predictability among polyploid lineages.

The *Spartina* genus (Poaceae, Chloridoideae) provides excellent opportunities to explore such processes at different evolutionary time scales, providing a phylogenetically well-understood framework of polyploidization that occurred in the last 10–12 my (Ainouche et al., 2012; Rousseau-Gueutin et al., 2015). Hybridization and polyploidy have recurrently shaped the history of this genus (also considered as a subsection in the *Sporobolus* genus; Peterson et al., 2014 but see Bortolus et al., 2019) and have resulted in modern species with various ploidy, ranging from tetraploids to dodecaploids, with a basic chromosome number of *x* = 10 (Marchant, 1968a; Ainouche et al., 2012). No diploid species was reported to date in this genus which most likely evolved from a tetraploid ancestral lineage (2*n* = 4*x* = 40) that diversified mainly in North and South America (and more recently in Europe) where they colonize coastal and inland marshes (Mobberley, 1956; Baumel et al., 2002a). In this study, we focused on seven species representing different ploidy, selected on the basis of their known phylogenetic history (Baumel et al., 2002a; Fortune et al., 2007; Rousseau-Gueutin et al., 2015). In tetraploids, two sister species were selected: *S. bakeri* and *Spartina patens*. *S. bakeri* (sand cordgrass) is native to the southeastern United States, where it grows on sandy beaches along the coast and in inland freshwater habitat in Florida and southern Georgia. It is morphologically highly similar to *S. patens* (saltmeadow grass), from which it can be distinguished by its unique vegetative habit (wanting rhizomes) and tolerance to freshwater (Mobberley, 1956). *S. patens* is distributed along the eastern United States to the Caribbean and northeast Mexico and has been introduced to the Mediterranean coast since the mid-19th century (Fabre, 1849) where it has been first considered as an endemic species and named *S. versicolor* Fabre. Recent molecular studies revealed that this latter actually represents an introduced, invasive genotype of *S. patens* (Prieto et al., 2011; Baumel et al., 2016). In the hexaploid clade, which diverged from the tetraploids 6–10 mya (Rousseau-Gueutin et al., 2015), we analyzed two species that diverged c.a. 2–4 mya: *S. alterniflora* (2*n* = 6*x* = 62) native to the Atlantic American coasts and *S. maritima* (2*n* = 6*x* = 60) native to the European Atlantic coast (Baumel et al., 2002a). During the 19th century, introductions of *S. alterniflora* to Europe led to hybridization with the native species and had important ecological and evolutionary consequences (reviewed in Ainouche et al., 2009; Strong and Ayres, 2013). Hybridization with *S. maritima* (as male parent) resulted in two natural F1 hybrids: *S.* × *townsendii* (2*n* = 6*x* = 62; Groves and Groves, 1880) in Southern England and *S.* × *neyrautii* (2*n* = 6*x* = 62; Foucaud, 1897) in south–west France (Marchant, 1963; Baumel et al., 2003; Ainouche and Wendel, 2014). Chromosome doubling of *S.* × *townsendii* at the end of the 19th century resulted in the formation of the new fertile allododecaploid (2*n* = 12*x* = 124) *S. anglica* (Hubbard, 1965; Marchant, 1968a). This system is now considered as one of the textbook examples of recent allopolyploid speciation, which allows examining the immediate effects of hybridization and genome duplication in natural populations (Ainouche et al., 2004).

Polyploidy and hybridization have important adaptive consequences in *Spartina*. Hexaploid species evolved anatomical and physiological traits [including salt and xenobiotic tolerance increased by specific cells such as salt glands or trichomes, production of DMSP (Dimethylsulfoniopropionate), an osmoprotectant molecule; Céccoli et al., 2015; Paredes-Páliz et al., 2017; Robertson et al., 2017; Rousseau et al., 2017; Cavé-Radet et al., 2019b] providing the ability to colonize low marsh zones and tolerate several hours of tidal immersion (Maricle et al., 2009; Bedre et al., 2016). Invasive abilities appear as an immediate consequence of hybridization and/or genome duplication in *Spartina* (Strong and Ayres, 2013; Ainouche and Gray, 2016). The neopolyploid *S. anglica* has rapidly expanded in range in its native region (Europe) and has been deliberately introduced in several continents for land reclamation and estuary stabilization (e.g., China, Australia, and New Zealand), where several attempts of eradication are being conducted to control the species (Gray and Benham, 1990; Hammond and Cooper, 2002; Ainouche et al., 2009). This recently formed allopolyploid species exhibits higher ecological amplitude and higher stress tolerance than its parental species (Cavé-Radet et al., 2019b). The highly duplicated nature of its allododecaploid genome is thought to play an important role in the plasticity and the observed adaptive features. First investigations of global gene expression using microarrays on this system revealed various patterns of non-additive parental expression affecting genes involved in stress tolerance (Chelaifa et al., 2010b; Ferreira de Carvalho et al., 2017). Significant DNA methylation changes were also observed notably in regions flanking transposable elements, as evaluated from methylation-sensitive AFLP (Salmon et al., 2005) and methylation sensitive transposon display (Parisod et al., 2009). More recently, reference transcriptomes were built using Next Generation Sequencing for the hexaploid and allododecaploid species (Ferreira de Carvalho et al., 2013b; Boutte et al., 2016) and small-RNAs (Cavé-Radet et al., 2019a) and repetitive sequences (Giraud et al., 2020) from tetraploid and hexaploid *Spartina* genomes were annotated.

**FIGURE 1:**
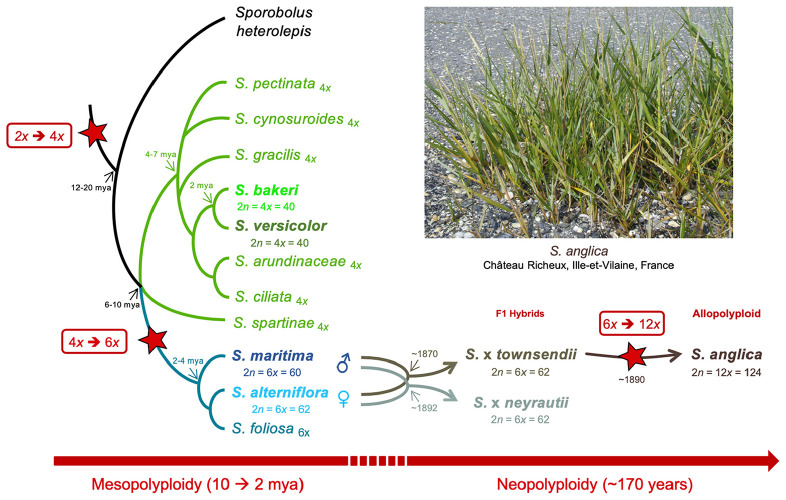
Phylogenetic relationships among *Spartina* species (redrawn from Baumel et al., 2002a), and position of the analyzed tetraploid (*Spartina bakeri* and *Spartina versicolor*), hexaploid (*Spartina maritima*, *Spartina alterniflora*, *Spartina* × *townsendii*, *Spartina* × *neyrautii*) and dodecaploid (*Spartina anglica*) taxa analyzed in this study. Divergence times (my) represent upper limits estimated so far (Rousseau-Gueutin et al., 2015). Stars indicate whole genome duplication (WGD) events during *Spartina* evolution.

In this study, gene and TE expressions were analyzed at different evolutionary time scales (Figure 1). The two sister tetraploid species and two hexaploid species were first compared to understand how gene and TE expressions evolved since their divergence 6–10 mya. Then, the evolution of gene and TE expression following recent hybridization and neopolyploidy was analyzed by comparing the species involved in the recent allopolyploid speciation, including the hexaploid parental species, their reciprocal F1 hybrids and the allododecaploid formed during the end of the 19^th^ century. More specifically, we addressed the following questions: (i) how similar are the tetraploid and hexaploid transcriptomes? (ii) how additive are F1 hybrids/allopolyploid transcriptomes compared to parental transcriptomes? (iii) how has the hybridization *vs.* genome duplication affected genes and the expression of TEs? (iv) are the transcriptomes of two independently formed hybrids affected in the same way? (v) what are the functions affected by interspecific hybridizations and/or genome duplication? To answer these questions, reference transcriptomes were assembled for each investigated species (providing new reference transcriptomes for the tetraploid species and enriching previously assembled transcriptomes of hexaploids and hybrid/allopolyploid species Ferreira de Carvalho et al., 2013b; Boutte et al., 2016). Homologous (putative orthologous) transcripts were identified among species and the relative contribution of homeologs was assessed for a subset of non-additively expressed genes in the hybrids and/or the allopolyploid.

## Materials and Methods

### Plant Material, RNA Extraction, and Sequencing

Among the seven *Spartina* species analyzed in this study (Figure 1), two individuals per species were collected from their natural habitat along the French coasts for *S. maritima*, *S. alterniflora*, *S.* × *neyrautii*, *S. anglica*, and *S. versicolor*, in Hythe (England) for *S.* × *townsendii* and in Florida (United States) for *S. bakeri* (Table 1). Previous transcriptome analyses (Ferreira de Carvalho et al., 2017) indicated that low intraspecific expression variation was encountered among plants collected along a tidal gradient in *Spartina* populations, but in order to limit potential plastic responses among samples, all individuals were transplanted to the same natural site (Morbihan, France on June, 19, 2013) in order to maintain all plants in the same environmental conditions. After 1 month of acclimation (2013/07/18), newly formed leaves from each individual were harvested between 10–12 am and flash frozen in liquid nitrogen. Thereafter, their RNAs were directly extracted according to Chelaifa et al. (2010a). Illumina libraries were prepared according to the Tru-Seq PCR-Free Protocol provided by Illumina. One Illumina library was prepared per individual (two individuals per species) and was sequenced on two independent lanes. The *S. anglica* libraries were sequenced twice more than the hexaploid species because of its larger genome (each individual was sequenced on 4 independent lanes; see Table 1 and Supplementary Table 1). Sequencing was performed at the BGI (Beijing Genomics Institute) using the Hi-Seq 2000 technology (100 bp paired-ends reads; insert-size of 500 bp). Raw sequence data have been deposited at GenBank under the accession number PRJNA657699.

**TABLE 1:** Studied polyploid *Spartina* species and summary of RNA-Seq Illumina datasets used.

****Species****	****Synonyms****	****Ploidy, number of chromosomes****	****Sample origin****	****Number of replicates****	****Total library depth (cumulative number of paired reads)****
***S. maritima*** Curtis	***Sporobolus maritimus*** (Curtis) P.M. Peterson & Saarela, comb. nov.	6*x* = 60	Le Hezo (Morbihan, France)	4	146,438,783
***S. alterniflora*** Loisel.	***Sporobolus alterniflorus*** (Loisel.) P.M. Peterson & Saarela, comb. nov.	6*x* = 62	Le Faou (Finistère, France)	4	179,792,458
***S. x townsendii*** H. Groves & J. Groves	***Sporobolus* × *townsendii*** (H. Groves & J. Groves) P.M. Peterson & Saarela, comb. nov.	6*x* = 62	Hythe (Hampshire, United Kingdom)	4	171,773,818
**S. × *neyrautii*** Foucaud	–	6*x* = 62	Hendaye (Pyrénées Atlantiques, France)	3	139,586,516
***S. anglica*** C. E. Hubb.	***Sporobolus anglicus*** (C.E.Hubb.) P.M. Peterson & Saarela, comb. nov.	12*x* = 124	Le Hezo (Morbihan, France)	8	296,374,022
***S. versicolor*** Fabre	***Sporobolus versicolor*** (Fabre) P.M. Peterson & Saarela, comb. nov.	4*x* = 40	Aresquiers (Hérault, France)	3	76,965,982
***S. bakeri*** Merr.	***Sporobolus bakeri*** (Merr.) P.M. Peterson & Saarela, comb. nov.	4*x* = 40	Florida (United States)	2	85,839,501

### Enrichment of Hexaploid Reference Transcriptomes and New Tetraploid Transcriptomes

Prior to differential gene expression analyses, the reference transcriptomes previously obtained by co-assembling pyrosequencing (Ferreira de Carvalho et al., 2013b) and Illumina data (Boutte et al., 2016) were enriched with the set of newly sequenced reads to build updated references for hexaploid and allododecaploid species (data used for reference transcriptome assemblies are available on GenBank, BioProject PRJNA338100). Considering the complex assembly process of transcriptomes in polyploid species, bioinformatics tools used were configured in order to obtain contigs representing consensus sequences of all expressed homeologous copies (or homeologs) from one given gene (Ferreira de Carvalho et al., 2013b). The expression level estimates for each contig was thus the sum of expressions of homeologs (and possibly, slightly divergent paralogs, resulting from recent individual gene duplication). Individual copies of a given gene were detected after remapping reads to these contigs, detecting SNPs (Single Nucleotide Polymorphisms) and phasing them to build haplotypes (see section Parental Origin of Transgressively Expressed Orthologs).

First, mappings of new Illumina reads on reference transcriptomes were performed using Bowtie2 (parameters: local alignment; authorized mismatch; G, 52, 8; Langmead and Salzberg, 2012). Approximately, 60% of reads per library were aligned on respective transcriptoms. Non-mapped reads, corresponding to the expression of non-assembled transcripts, were then pooled by species and assembled following a method previously described (Boutte et al., 2016). Briefly, reads were assembled using the Trinity algorithm (v. 2.8.4; Grabherr et al., 2011) with a *k*-mer size of 25 bp and a minimum assembled contig length of 48 bp. To improve the assembly, all new contigs with a length higher than 100 bp were co-assembled with Newbler software (parameters: ml = 40 bp; mi = 90%; v.2.8 Margulies et al., 2005). Finally, a selfBLAST of all new contigs was performed using BLAST+ (v. 2.5.0; Altschul et al., 1990) to identify redundant sequences. Contigs included in other contigs were removed and contigs with a minimum overlapping of 50 bp, with an identity higher or equal to 90% were assembled using custom python scripts (Boutte et al., 2016). The newly assembled contigs were then added to previous reference transcriptomes (Boutte et al., 2016) for each hexaploid species (*S. maritima*, *S. alterniflora*, *S.* × *townsendii*, and *S.* × *neyrautii*) and the allopolyploid *S. anglica*. As no reference transcriptomes were available for the analyzed tetraploid species, Illumina libraries sequenced for expression analyses were used to assemble *de novo* reference transcriptomes for each tetraploid species. Reads were assembled using the method described above (Trinity assembly followed by Newbler co-assembly and custom scripts to remove redundancy). Quality of assemblies was tested using BUSCO (v. 4.1.4; Seppey et al., 2019). Transcriptome completeness was measured by comparing new assembled transcriptomes with pre-selected BUSCO genes specific to Poales species (lineage dataset: poales_odb10; creation date: 2020-08-05; number of species: 12; number of BUSCO genes: 4,896; *e*-value < 1e-06).

### Functional Annotations

Prior to contig annotations, potential contamination during RNA extraction and/or library sequencing was verified using BLASTn (Altschul et al., 1990) against the NCBI nucleotide database (accessed 08/12/2018). Such contigs displaying similarities with Prokaryote, Fungi, Protist or Metazoan sequences were removed from the analyses (*e*-value < 1e-6 and 90% of identity).

New assembled and reference transcriptomes were annotated as detailed in Ferreira de Carvalho et al. (2013b) and Boutte et al. (2016). Contigs were aligned via BLASTx analyses (one-way Best Blast Hits with an *e*-value < 1e-6) against protein databases from six species: *Arabidopsis thaliana* (genome version TAIR10), *Oryza sativa* (v. IRGSP-1.0), *Brachypodium distachyon* (v. 3.0), *Panicum hallii* (v. 3.1), *Sorghum bicolor* (v. 3), *Zea mays* (v. B73 RefGen v4). Gene Ontologies (GO terms) of each gene identified in our transcriptomes were retrieved from plant protein databases and included in our annotations. Similarities with known protein domains were also found using hmmer [a Hidden Markov Model (HMM) algorithm hmmer.org, v. 3.2] against the Pfam database (v. 32.0 Finn et al., 2014). Figures representing results from annotation analyses were drawn using the VennDiagram (v. 1.6.20; Chen and Boutros, 2011) and the UpSetR v. 1.4 R packages (Conway et al., 2017).

To complete annotations, contigs were also aligned against the *Spartina* transposable element databases built by Giraud et al. (2020) as well as against ribosomal DNA and chloroplast genomes previously assembled (Boutte et al., 2015; Rousseau-Gueutin et al., 2015) using BLASTn (*e*-value < 1e-6; Altschul et al., 1990) and Bowtie2 (parameters: local alignment; authorized mismatch; G, 52, 2; Langmead and Salzberg, 2012).

### Identification of Orthologous Transcripts/Regions Among Species

To compare gene expression among *Spartina* species, the OrthoVenn2 program (Xu et al., 2019) was used to identify orthologous contigs or (orthologs) in the different transcriptomes. This program provides clusters of orthologous sequences by sequence comparison analyses (BLASTp) and graph-based clustering among up to eight plant species simultaneously. In order to reduce the computational time, only annotated contigs were conserved for analyses. Prior to OrthoVenn2 analysis, contigs were translated into protein sequences (in the 6 reading frames) using the EMBOSS transeq tool (v. 6.6.0; Madeira et al., 2019). OrthoVenn2 was run with default parameters (*e*-value 1e-5; inflation value 1.5) to identify orthologs among the different transcriptomes. OrthoVenn2 allowed grouping orthologs found in the studied species into the same cluster. Within each cluster, regions or windows where orthologs showed sequence similarities were delimited in order to measure expression levels in each species from the same sequence length. To achieve this, all contigs or orthologs within the same cluster were aligned against each other using BLASTn (*e*-value < 1e-6; Altschul et al., 1990) and windows displaying a maximal number of aligned contigs and a maximal size were selected using custom python scripts for differential expression analyses.

### Differential Expression Analyses

Gene expression levels were measured by mapping reads of each sequenced library on newly assembled reference transcriptomes using the Bowtie2 software with the following parameters: local alignment; authorized mismatch; G, 52, 8 corresponding to 90% of minimum identity (Langmead and Salzberg, 2012). For each cluster of orthologs, gene expression levels were given by the number of reads mapped on contigs and included in windows or regions previously selected.

Expression of TEs was also estimated according to the same method as used for gene expression. RNA-seq libraries were mapped using Bowtie2 (same settings as mentioned above) on their respective *Spartina* TE database previously built by Giraud et al. (2020). The expression of each TE family was measured by the total number of reads aligned on the full-length sequence of the elements.

Read counts for genes and TEs were calculated separately for each sequenced library representing biological or technical replicates. Data counts were extracted with samtools (v. 1.3.1; Li et al., 2009) and were normalized using the EDAseq Bioconductor R-package (v. 2.18.0; Risso et al., 2011) in order to correct sequencing bias within each library (GC-content and gene length) and between libraries (sequencing depth) (Bullard et al., 2010; Li et al., 2015). These biases were corrected according to the full-quantile normalization implemented in EDASeq. Normalization factor with DESeq2 was skipped because differences in sequencing depth were already taken into account with EDASeq. After normalization, data quality assessment and sample homogeneity were analyzed by clustering (Euclidean distances between samples) and principal component analysis (PCA).

Statistical analyses of gene and TE expression were performed with the DESeq2 package (Love et al., 2014). This package calculates log2 fold-change between species (from normalized count), estimates the dispersion and uses Wald tests (negative binomial linear model) to determine significant differential expression. Before DESeq2 analyses, clusters of orthologs weakly expressed in all studied species (mean expression of all replicates lower than 5 reads after normalization step) were removed because (i) they potentially represented noise from RNA sequencing and (ii) their low counts could affect the statistical tests (biased dispersions and variance in log2 fold change). Regarding the limited number of biological replicates per species we considered for each species both biological and technical replicates without distinction, in order to maximize the intraspecific variance. Considering the large number of tests, adjusted p-values were estimated according to Bonferroni correction to minimize false-positive rates. In our analyses, genes and TEs were considered as differentially expressed (DE) between two species when the adjusted *p*-value was lower than 0.01.

Several comparisons were performed: (i) expression levels between species presenting the same ploidy or of the same hybrid origin (*S. versicolor* vs. *S. bakeri*; *S. alterniflora* vs. *S. maritima*; *S.* × *townsendii* vs. *S.* × *neyrautii*), (ii) expression levels between the two tetraploid species (*S. versicolor* vs. *S. bakeri*) and the two hexaploid species (*S. alterniflora* vs. *S. maritima*), (iii) expression levels between each F1 hybrid (*S.* × *townsendii* vs. *S.* × *neyrautii*) and the MPV (Mid Parent Value; expected under additive parental expression) and (iv) expression levels between *S.* × *townsendii* and *S. anglica*. The MPV was calculated by averaging the expression levels found in both parental species (mean of expression levels found in all parental replicates after normalization). Genes that deviate from parental additivity in hybrids and allopolyploid (statistically DE compared to the estimated MPV) were classified in different patterns: under parental dominance when the gene expression level found in the hybrids or allopolyploid was not statistically DE compared to the expression level found in the maternal or paternal species (maternal or paternal dominance, respectively); transgressively expressed when the gene expression level found in the hybrids or allopolyploid was statistically DE compared to the expression levels found in maternal and in paternal species (transgressively up-regulated or down-regulated).

### GO Enrichment Analysis

Gene ontology analyses were conducted to identify metabolic pathways or biological processes that were found differentially expressed following recent hybridization and genome doubling or between tetraploid and hexaploid species. GO term enrichment analyses were performed with the online platform agriGO (v. 2.0; Tian et al., 2017), considering the GO terms associated with *A. thaliana* (GO annotations available on TAIR10). The set of reference genes included only a subset of Arabidopsis genes for which homologous genes were found in *Spartina* transcriptomes. Biological processes over-represented in DE genes were determined using hypergeometric statistical tests using Singular Enrichment Analysis tool (Bonferroni multi-test adjustment, *p*-value < 0.01).

### Parental Origin of Transgressively Expressed Orthologs

We selected a set of non-additive, transgressively expressed orthologs in the F1 hybrid *S.* × *townsendii* and/or the allopolyploid *S. anglica* compared to their parental species (*S. alterniflora* and *S. maritima*) and identified the parental origin of the transcribed hybrid/allopolyploid haplotypes (homeologous copies) involved in such an expression pattern. The detection of haplotypes was performed as previously described for high ploidy-level species without diploid genome reference (Boutte et al., 2016) by detecting polymorphisms (in regions with minimum read-depth of 30; with minimum proportion of reads displaying the alternative character state of 10%) and phasing reads sharing at least two character states in sliding windows of 120 bp.

## Results

### New Reference Transcriptomes and Functional Annotations

The *de novo* transcriptome assembly process allowed building of new reference transcriptomes for the two tetraploid species containing 103,101 contigs for *S. versicolor* and 107,319 contigs for *S. bakeri*. Non-aligned reads to previously assembled transcriptomes of the hexaploid and allododecaploid species (40% of reads) were separately assembled, which resulted in the enrichment of reference transcriptomes from 158,825 to 240,710 contigs according to species (Table 2). Consequently, the new reference transcriptomes used for expression analyses contained 217,800 and 206,348 contigs for the parental hexaploid species *S. maritima* and *S. alterniflora*, respectively, 281,153 and 266,906 contigs in the F1 hybrids *S.* × *townsendii* and *S.* × *neyrautii*, and 297,327 contigs in the allododecaploid *S. anglica*. Their GC-contents range from 45 to 49% and their N50 varies from 417 bp in the F1 hybrids and the allopolyploid to 724 bp in the tetraploid species, with an intermediate value in the hexaploid parental species (471–494 bp). Quality of the new reference transcriptomes was assessed using BUSCO with a panel of genes representing Poales species. Results are given in Supplementary Figure 1.

**TABLE 2:** *Spartina* reference transcriptomes and functional annotations.

**Species**	***S. maritima***	***S. alterniflora***	***S.* × *townsendii***	***S.* × *neyrautii***	***S. anglica***	***S. versicolor***	***S. bakeri***
Number of contigs in reference transcriptomes (Boutte et al., 2016)	58,975	43,521	58,120	62,101	56,617	–	–
Number of new assembled contigs	158,825	162,827	223,033	204,805	240,710	103,101	107,319
Total number of contigs in reference transcriptome	217,800	206,348	281,153	266,906	297,327	103,101	107,319
Number of annotated contigs	76,916 (35%)	69,980 (34%)	93,645 (33%)	91,798 (34%)	94,130 (32%)	66,924 (65%)	62,827 (59%)
Number of unigenes (detected using the *O. sativa* genome)	22,186	22,133	23,756	23,139	23,544	19,328	19,779
GC-content	45.18%	45.25%	46.10%	45.43%	45.33%	49.30%	47.23%
N50 (bp)	471	494	418	417	417	700	724
Mean contig length (bp)	345	351	313	322	315	559	565
Median contig length (bp)	230	226	211	223	209	358	356
Minimum contig length (bp)	100	100	100	100	100	100	100
Maximum contig length (bp)	10,313	10,839	9,423	14,705	8,833	17,112	16,705

Functional annotations were assigned to 35 and 34% of contigs in *S. maritima* and *S. alterniflora*, respectively (Table 2). The vast majority of annotations was performed with the Blastx method (e.g., in *S. maritima*, Blastx allowed us to assign a function to 86% of annotated contigs and 48% with the HMM-Pfam method; Supplementary Figure 2). In comparison with parental species, the same proportions of contigs were also annotated in the F1 hybrids and the allopolyploid with 93,645 contigs (33%) in *S.* × *townsendii*, 91,798 contigs (34%) in *S.* × *neyrautii* and 94,130 contigs (32%) in *S. anglica*. Finally, within tetraploid transcriptomes, 65% of contigs in *S. versicolor* and 59% of contigs in *S. bakeri* were annotated (representing, respectively, 66,924 and 62,827 contigs). In order to reduce the computational time of analyses, we explored gene expression evolution for this set of annotated contigs, which represent a large part of the global gene expression in *Spartina* leaves (83% of mapped reads for each library Supplementary Table 2).

### Orthologous Contig Identification

**FIGURE 2:**
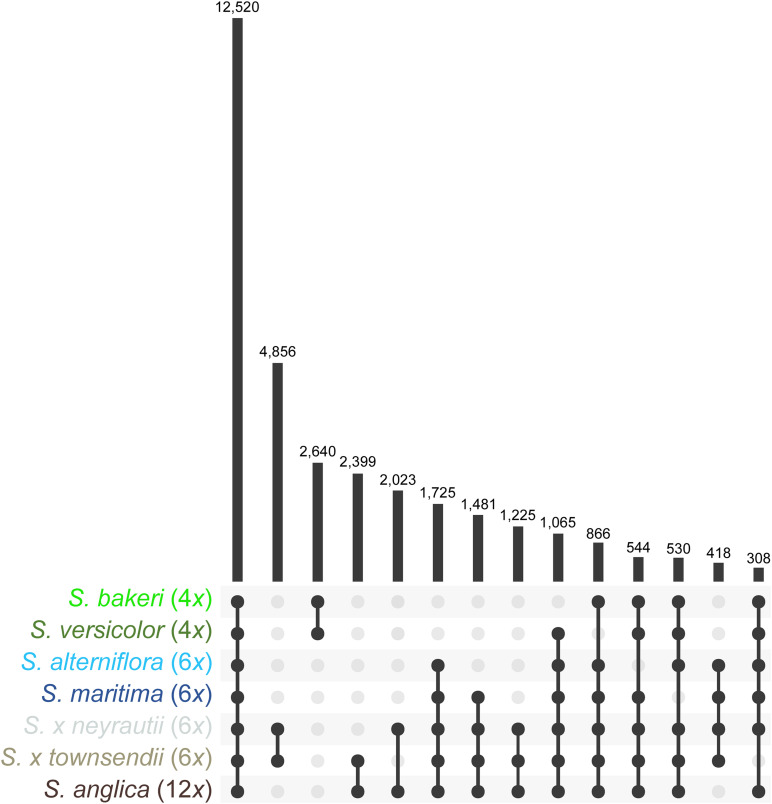
Orthologs found within reference transcriptomes of *Spartina* species. Values represent the number of clusters retrieved in two or more species.

Orthologous contigs among reference transcriptomes were identified and 62,830 clusters of orthologs were formed using OrthoVenn2. It is important to note that a few clusters may sometimes belong to a single gene if it could not be fully assembled. Among these clusters, 12,520 contained at least one ortholog of each of the seven *Spartina* species, 1,725 contained only orthologs of hexaploid species and their derived taxa (orthologs found in transcriptomes of the parental species, the F1 hybrids and the allododecaploid) and 2,640 contained only orthologs of tetraploid species (Figure 2). In addition, 4,856 clusters contained exclusively orthologs identified in both F1 hybrids, and 2,399 orthologs of only *S.* × *townsendii* and *S. anglica*. Overall, the identification of orthologs with the OrthoVenn2 method allowed finding orthologs for more than 62% of annotated contigs [from 62% in *S. versicolor* (41,832 contigs) to 66% in *S. alterniflora* (50,245 contigs); Supplementary Table 2]. The composition of each cluster of orthologs (contig name, annotation) is detailed in Supplementary Table 3.

### Gene Expression

After data normalization and elimination of low counts, 42,423 clusters of orthologs were retained for statistical analyses (representing 19,417 unigenes detected using the *O. sativa* genome). Data quality was assessed via hierarchical clustering (Euclidean distances) and principal component analysis of counts from each library (Supplementary Figure 3). Given the absence of aberrant results, all libraries from each species were conserved as replicates for analyses.

#### Expression Evolution in Tetraploid and Hexaploid Contexts

**FIGURE 3:**
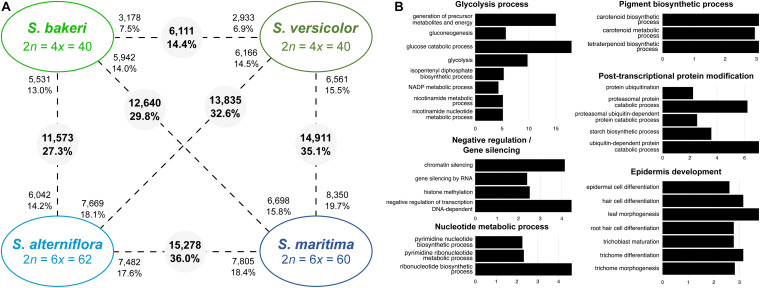
Transcriptomic changes between tetraploids species (*S. bakeri* and *S. versicolor*) and hexaploid species (*S. alterniflora* and *S. maritima*). **(A)** Number of differentially expressed genes between species (pairwise comparisons). The numbers in grey circles represent the number of orthologs differentially expressed among the 42,423 analyzed clusters. The numbers of upregulated orthologs are represented near species names. **(B)** Bar graph of the most enriched GO-terms (biological process) for the differentially expressed orthologs between tetraploid and hexaploid species. GO-terms were grouped according to their metabolic pathway affiliation. *X*-axis corresponds to the negative logarithm of adjusted *p*-value. The complete list of enriched GO terms is available in Supplementary Tables 4, 5.

Comparisons between tetraploids showed that in leaves, 6,111 contigs (or orthologs) were significantly differentially expressed (DE) between *S. versicolor* and *S. bakeri*, representing 14.4% of the studied contigs (Figure 3A). Almost half of them were over-expressed in *S. bakeri* (3,178; 7.5%) the other half being over-expressed in *S. versicolor* (2,933; 6.9%). Orthologs more expressed in *S. bakeri* appear to be involved in cell development, modification and protein transport, whereas orthologs more expressed in *S. versicolor* are involved in epidermis development, response to biotic and abiotic stresses (salt, light, cold, and bacterium), gene silencing and post-transcriptional protein modifications (Supplementary Table 4).

More orthologs (15,278; 36.0%) were found DE between the hexaploid species *S. maritima* and *S. alterniflora* than between the tetraploids. Among them, 7,482 orthologs (48.9% of DE contigs) were over-expressed in *S. alterniflora* compared to *S. maritima* and were mainly involved in cell development, post-translational protein modifications (protein desumoylation, protein amino acid myristoylation), fatty acid and starch metabolic processes, response to salt and cold stresses and gene silencing (Supplementary Table 4). Conversely, 7,805 orthologs (51.1% of DE contigs) were over-expressed in *S. maritima* and were involved in chloroplast and epidermis development, protein catabolic process, post-translational protein modifications (amino acid phosphorylation and methylation), glycolysis process. When comparing tetraploids with hexaploids, the number of DE orthologs varies from 11,573 (27.3%) between *S. bakeri* and *S. alterniflora* to 14,911 (35.1%) between *S. versicolor* and *S. maritima* (Figure 3A). GO-term enrichment analyses showed that these DE orthologs have notably a key role in glycolysis process, epidermis development, pigment biosynthetic process and gene silencing (Figure 3B and Supplementary Table 5).

#### Expression Evolution Following Recent Hybridization Events

**FIGURE 4:**
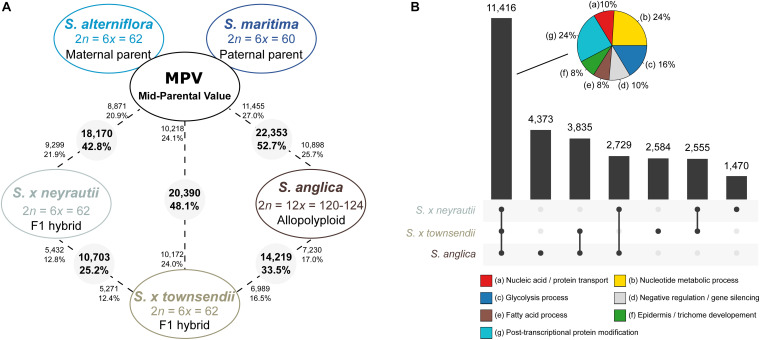
Transcriptomic changes in F1 hybrids and the allopolyploid species compared to the *in silico* MPV (mid-parent value). **(A)** The number (and percentages) of differentially expressed (DE) genes between the MPV (expression expected under additive parental expression) and the F1 hybrids (*S.* × *townsendii* and *S.* × *neyrautii*) or the allopolyploid *S. anglica* are provided. The numbers in grey circles represent the number of orthologs differentially expressed among the 42,423 analyzed clusters. The numbers of upregulated orthologs are represented near species names. **(B)** Diagram of common DE genes between the MPV and species deriving from recent allopolyploidization. Pie charts represent enriched metabolic pathways within 11, 416 genes found DE compared to the MPV both among *S.* × *townsendii, S.* × *neyrautii*, and *S. anglica* (% of genes associated with enriched metabolic pathways). The complete list of enriched GO terms is available in Supplementary Table 6.

In order to examine the transcriptomic effects of interspecific hybridization in the two independently formed F1 hybrids (with *S. alterniflora* as maternal parent and *S. maritima* as paternal parent), gene expression levels were compared first with the *in silico* MPV (average expression between parental species) (Figure 4A). Then orthologs that were found differentially expressed between the two hybrids were examined to (i) identify the potentially affected functions and (ii) in which way these DE genes deviate from parental additivity.

Comparisons of each hybrid with the MPV revealed that among 42,423 studied clusters of orthologs, 48.1% of clusters (20,390 orthologs) in *S.* × *townsendii* and 42.8% of clusters (18,170 orthologs) in *S.* × *neyrautii* deviated from parental additivity. For each hybrid, an equivalent number of DE orthologs was under-expressed compared to the MPV (10,218 and 8,871 orthologs, respectively in *S.* × *townsendii* and *S.* × *neyrautii*) and over-expressed compared to the MPV (10,172 and 9,299 orthologs, respectively in *S.* × *townsendii* and *S.* × *neyrautii*). Expression patterns in hybrids (Table 3) revealed that 70% of non-additive genes were expressed similarly to one of the parental species (parental expression dominance). In *S.* × *townsendii*, 6,908 orthologs (16.3%) mimicked the maternal parent (*S. alterniflora*) whereas 7,151 orthologs (16.9%) mimicked the paternal parent *S. maritima*. In *S.* × *neyrautii*, 6,421 orthologs (15.1%) mimicked the maternal parent *S. alterniflora* whereas 6,466 orthologs (15.2%) mimicked the paternal parent *S. maritima*. Other DE genes were found transgressively expressed in the hybrids compared to the MPV. Interestingly, in both hybrids a higher number of orthologs was more transgressively up-regulated (3,884 and 3,452 in *S.* × *townsendii* and *S.* × *neyrautii*, respectively) than transgressively down-regulated (2,447 and 1,831, respectively).

**TABLE 3:** Gene expression patterns in the two F1 hybrids and the allopolyploid compared to the parental species *S. alterniflora* (the maternal parent) and *S. maritima* (the paternal parent).

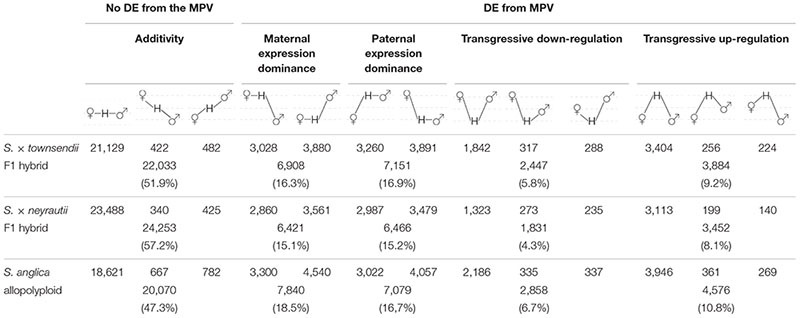

*For each taxon, the first line indicates the number of contigs from each pattern, the second line the total number of contig from each category and the third line indicates the percentage of contigs from each category.*

**FIGURE 5:**
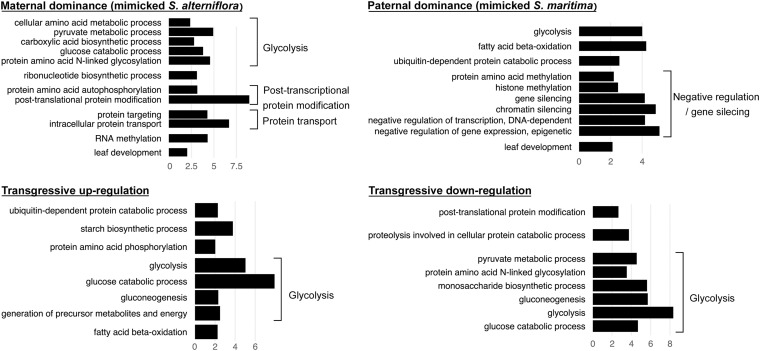
Bar graph of the most enriched GO-terms (biological process) corresponding to genes found DE compared to the MPV and with the same expression patterns in both F1 hybrids *S.* × *townsendii* and *S.* × *neyrautii* (e.g., bar graph of maternal dominance represents the enriched functions of the 3,045 genes found under maternal dominance in *S.* × *townsendii* and in *S. neyrautii*). GO-terms were grouped according to their metabolic pathway affiliation. *X*-axis corresponds to the negative logarithm of adjusted *p*-value.

We found that among the genes deviating from parental additivity in hybrids (20,390 orthologs in *S.* × *townsendii* and 18,170 orthologs *S.* × *neyrautii* as indicated above), 13,971 of them were DE compared to MPV in both hybrids (Figure 4B). These genes were mainly involved in post-transcriptional protein modification processes, nucleotide metabolic processes and glycolysis (Figure 4B and Supplementary Table 6). Enriched GO-terms also belongs to gene silencing mechanisms (e.g., GO:0006342 chromatin silencing; GO:0016571 histone methylation; GO:0035196 production of miRNAs involved in gene silencing by miRNA). More specifically, 63% of DE orthologs (8,848/13,971) shared a similar pattern in both hybrids: 6,322 orthologs were under the same parental dominance and 2,526 orthologs were transgressively expressed in *S.* × *townsendii* and *S.* × *neyrautii* (Table 4). Enriched GO-terms for genes under paternal dominance in *S.* × *townsendii* and *S.* × *neyrautii* were mainly involved in gene silencing processes whereas genes under maternal dominance were involved in glycolysis process, post-transcriptional protein modification and protein transport (Figure 5). Other genes involved in the glycolysis process also appeared transgressively expressed in both F1 hybrids.

**TABLE 4:** Gene expression pattern evolution in the two independently formed hybrids *S.* × *townsendii* and *S.* × *neyrautii*.

**Expression pattern in *S.*** × ***townsendii***	**Expression pattern in *S.*** × ***neyrautii***	**Number of genes**	**% of genes**
**Additive**	Additive	17,834	**42.0%**
	Maternal expression dominance	1,069	2.5%
	Paternal expression dominance	984	3.3%
	Transgressive down-regulation	638	1.5%
	Transgressive up-regulation	1,508	3.6%

**Maternal**	Additive	1,730	4.1%
**expression**	Maternal expression dominance	3,045	**7.2%**
**dominance**	Paternal expression dominance	1,826	4.3%
	Transgressive down-regulation	155	0.4%
	Transgressive up-regulation	152	0.4%

**Paternal**	Additive	1,733	4.1%
**expression**	Maternal expression dominance	1,868	4.4%
**dominance**	Paternal expression dominance	3,277	**7.7%**
	Transgressive down-regulation	163	0.4%
	Transgressive up-regulation	110	0.3%

**Transgressive**	Additive	1,150	2.7%
**down-regulation**	Maternal expression dominance	216	0.5%
	Paternal expression dominance	209	0.5%
	Transgressive down-regulation	858	**2.0%**
	Transgressive up-regulation	14	0.1%

**Transgressive**	Additive	1,806	4.3%
**up-regulation**	Maternal expression dominance	223	0.5%
	Paternal expression dominance	170	0.4%
	Transgressive down-regulation	17	0.1%
	Transgressive up-regulation	1,668	**3.9%**

*The percentage of genes that conserved the same expression pattern in both hybrids are in bold.*

When comparing the two hybrids, we identified 10,703 DE orthologs (25.2% of clusters) between *S.* × *townsendii* and *S.* × *neyrautii* (Figure 4A). A large majority of these 25.2% orthologs (10,037; 93.8%) exhibits different expression patterns in each hybrid with regard to parental additivity (Supplementary Table 7). Specifically, 3,321 orthologs (31% of DE orthologs) were under parental dominance in both hybrids but did not mimic the same parent: 1,639 orthologs appeared under maternal dominance in *S.* × *townsendii* but under paternal dominance in *S.* × *neyrautii* and conversely, 1,682 orthologs appeared under paternal dominance in *S.* × *townsendii* and under maternal dominance in *S.* × *neyrautii*. These orthologs were involved in trichome development and response to stresses (salt, light, and cold) (Supplementary Table 7). Other DE orthologs between hybrids were mainly additive in one hybrid and expressed transgressively in the other hybrid (4,034 orthologs corresponding to 38% of DE orthologs). In total, 2,711 orthologs (25.3% of DE contigs) were transgressively up-regulated in one hybrid and additive in the other and 1,323 orthologs (12.4% of DE orthologs) were transgressively down-regulated in one hybrid and additive in the other (Supplementary Table 7). GO-term enrichment analyses reveal that orthologs transgressively up-regulated in *S.* × *townsendii* and additive in *S.* × *neyrautii* (1,378 orthologs) were involved in biotic/abiotic stress responses (virus, bacterium, salt, and cadmium) and in gene silencing (production of small RNA involved in gene silencing by RNA). In comparison, orthologs transgressively up-regulated in *S.* × *neyrautii* and additive in *S.* × *townsendii* (1,333 orthologs) were involved in gravitropism, cellular development and also in responses to abiotic stresses (salt and cadmium).

#### Expression Evolution Following Recent Allododecaploid Speciation

**FIGURE 6:**
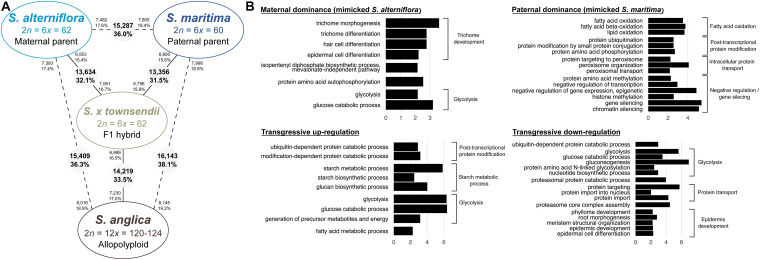
Transcriptomic changes following hybridization and whole genome duplication in *Spartina*. **(A)** Number of differentially expressed genes between species (pairwise comparisons). The numbers in grey circles represent the number of orthologs differentially expressed among the 42,423 analyzed clusters. The numbers of upregulated orthologs are represented near species names. **(B)** Bar graph of the most enriched GO-terms (biological process) for non-additively expressed orthologs in the F1 hybrid and in the allopolyploid (expressed under parental dominance or transgressive). GO-terms were grouped according to their metabolic pathway affiliation. The *X*-axis corresponds to the negative logarithm of adjusted *p*-value. The complete list of enriched GO terms is available in Supplementary Tables 6, 7.

Gene expression in *S. anglica* was compared (i) with the MPV and (ii) following the genome duplication *per se* by comparing gene expression of *S. anglica* with *S.* × *townsendii*. Analyses showed that 22,353 orthologs (52.7% of clusters) deviated from parental additivity in the allopolyploid, which is more than what was found in *S.* × *townsendii* (20,390 DE orthologs, 48.1% from MPV) (Figure 4A). The comparison against MPV revealed that 68% of DE orthologs in *S. anglica* were also DE in *S.* × *townsendii* (Figure 4B). These genes were mainly involved in glycolysis and fatty acid process, post-transcriptional protein modification process, nucleotide metabolic process and gene silencing (Figure 4B and Supplementary Table 6). As reported for the F1 hybrids, the majority of identified DE orthologs (67%) were under parental expression dominance (70% in *S.* × *townsendii*; Table 3). However, in *S. anglica*, the number of genes under maternal dominance was superior to genes under paternal dominance (7,840 orthologs expressed similarly as *S. alterniflora* against 7,079 expressed similarly as *S. maritima*). This is in accordance with species-species comparisons (Figure 6A), which showed a smaller number of DE orthologs between *S. anglica* and *S. alterniflora* (15,409; 36.3%) than between *S. anglica* and *S. maritima* (16,143; 38.1%). In addition, gene expression patterns revealed an increase of transgressively expressed orthologs in *S. anglica* (7, 434) compared to both F1 hybrids (6,331 in *S.* × *townsendii* and 5,283 in *S.* × *neyrautii*), with a large majority of them (62%) being transgressively up-regulated.

We found that 56.7% of the studied orthologs in *S. anglica* conserved the same pattern as the F1 hybrid after genome doubling (Table 5). Among these, 35.02% of the analyzed orthologs were additive in *S.* × *townsendii* and conserved the additivity in the allopolyploid *S. anglica*. Interestingly, in the F1 hybrid and in the allopolyploid, genes mimicking the expression found in the maternal parent *S. alterniflora* (3,074 orthologs; 7.2%) were mainly involved in trichome development (Table 5 and Figure 6B). Conversely, genes expressed as the paternal parent *S. maritima* (3,050 orthologs; 7.2%) were involved in gene silencing and down-regulation processes. Among genes expressed transgressively in *S.* × *townsendii* and *S. anglica*, those involved in epidermis development appear downregulated whereas those involved in starch metabolism appear upregulated.

**TABLE 5:** Evolution of gene expression patterns between the F1 hybrid *S.* × *townsendii* and the allopolyploid *S. anglica*.

**Expression pattern in *S.*** × ***townsendii***	**Expression pattern in *S. anglica***	**Number of genes**	**% of genes**
**Additive**	Additive	14,931	**35.2%**
	Maternal expression dominance	2,140	5.0%
	Paternal expression dominance	1,693	4.0%
	Transgressive down-regulation	1,208	2.8%
	Transgressive up-regulation	2,061	4.9%

**Maternal**	Additive	1,384	3.3%
**expression**	Maternal expression dominance	3,074	**7.2%**
**dominance**	Paternal expression dominance	1,896	4.5%
	Transgressive down-regulation	268	0.6%
	Transgressive up-regulation	286	0.7%

**Paternal**	Additive	1,455	3.4%
**expression**	Maternal expression dominance	2,109	5.0%
**dominance**	Paternal expression dominance	3,050	**7.2%**
	Transgressive down-regulation	268	0.6%
	Transgressive up-regulation	269	0.6%

**Transgressive**	Additive	795	1.9%
**down-regulation**	Maternal expression dominance	284	0.7%
	Paternal expression dominance	256	0.6%
	Transgressive down-regulation	1,081	**2.5%**
	Transgressive up-regulation	31	0.1%

**Transgressive**	Additive	1,505	3.5%
**up-regulation**	Maternal expression dominance	233	0.5%
	Paternal expression dominance	184	0.4%
	Transgressive down-regulation	33	0.1%
	Transgressive up-regulation	1,929	**4.5%**

*The percentage of genes that conserved the same pattern of expression in both species are in bold.*

When examining gene expression evolution following genome duplication *per se*, 14,219 orthologs (33.5%) were found DE between the F1 hybrid *S.* × *townsendii* and the allopolyploid *S. anglica* (Figure 6A). An equivalent number of genes were up-regulated in each species: 6,989 orthologs were over-expressed in *S.* × *townsendii* and 7,230 orthologs were over-expressed in *S. anglica*. Genes more expressed in *S.* × *townsendii* appeared involved in chloroplast development, sulfur biosynthetic process, glycolysis process and response to stress (water deprivation, bacterium, and fungus infection) whereas genes more expressed in *S. anglica* appeared involved in epidermis development, negative regulation/gene silencing, response to salt stress (Supplementary Table 8). Most of the expression changes affected (i) genes that were additively expressed in *S.* × *townsendii* and found under maternal dominance or transgressively up-regulated in *S. anglica* (5.0% and 4.9% of orthologs, respectively) or, (ii) genes that were under paternal dominance in *S.* × *townsendii* and found under maternal dominance in *S. anglica* (5.0%) (Table 5). Finally, more genes shifted toward maternal dominance in *S. anglica* (7,840 orthologs) than to paternal dominance (7,079 orthologs).

Detection of haplotypes was performed for a set of 58 orthologous contigs showing an expression level clearly different following hybridization and duplication (transgressively expressed in *S.* × *townsendii* and *S. anglica*) and with enriched functions. Among these 58 contigs, eight were too weakly expressed in the parents, preventing the identification of parental haplotypes in hybrids/allopolyploids; five were displaying no polymorphism within and between species; 28 were displaying an additive pattern (in terms of presence/absence) of parental haplotypes in the hybrids and the allopolyploid (but expressed in a transgressive manner); five were non-additive in *S.* × *neyrautii*, six in *S.* × *townsendii*; one in both hybrids; and five were non-additive in both F1 hybrids and the allopolyploid (three of them are outlined in Table 6). The gene encoding the subunit SSRP1 of the FACT complex (Facilitates Chromatin Transcription) was transgressively upregulated in *S.* × *townsendii* and *S. anglica*. For this gene, only the haplotype originating from *S. alterniflora* was detected in the F1 hybrid and the allopolyploid, together with haplotypes shared by the two parents (i.e., of “undetermined origin”). The two other genes involved in salt stress response, showed different contributions of the parental copies in their transgressive over-expression in the F1 hybrid and in the allopolyploid. For the CLC-d gene encoding a chloride channel protein, all haplotypes retrieved in the parental species were detected in *S.* × *townsendii* and *S. anglica*. However, for the gene encoding the salt-stress inducible tonoplast aquaporin 2, only haplotypes inherited from *S. maritima* were detected in *S.* × *townsendii*, whereas in *S. anglica*, haplotypes from both parental species were detected.

**TABLE 6:** Origin of haplotypes identified for three genes transgressively up-regulated following hybridization and genome doubling in *S.* × *townsendii* and *S. anglica.*

**Haplotype origin**	**Haplotypes inherited from *S. alterniflora***	**Haplotypes inherited from *S. maritima***	**Undetermined parental origin (with no polymorphism)**
**Subunit SSRP1 of FACT complex**			
Haplotypes identified in parental species	✔ (2)		✔ (1)
Origin of haplotypes identified in *S.* × *townsendii* (F1 hybrid)	✔ (2)		
Origin of haplotypes identified in *S. anglica* (Allopolyploid)	✔ (2)		
**Chloride channel protein CLC-d**			
Haplotypes identified in parental species	✔ (6)		✔ (5)
Origin of haplotypes identified in *S.* × *townsendii* (F1 hybrid)	✔ (3)		✔ (3)
Origin of haplotypes identified in *S. anglica* (Allopolyploid)	✔ (4)		✔ (4)
**Salt-stress inducible tonoplast intrinsic protein 2**			
Haplotypes identified in parental species	✔ (1)	✔ (2)	✔ (1)
Origin of haplotypes identified in *S.* × *townsendii* (F1 hybrid)		✔ (1)	
Origin of haplotypes identified in *S. anglica* (Allopolyploid)	✔ (1)	✔ (2)	

*The number of haplotypes detected is indicated in brackets.*

### TE Expression

**FIGURE 7:**
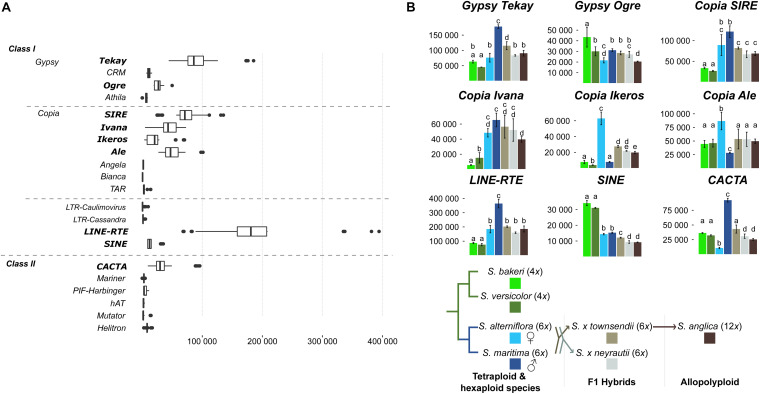
Expression of transposable elements. **(A)** Expression levels comparison between all TEs identified in the *Spartina* genomes (Giraud et al., 2020). Boxplots were calculated from TE expression levels (normalized reads count) considering each library of all studied species. **(B)** Variation of TE expression levels between species. Only the nine highest expressed TEs are shown (*p*-value < 0.01). Letters above bars (from **a**–**e**) indicate significant differences of TE expression between species.

A strong transcriptional activity was detected for nine TE families in the analyzed *Spartina* species (Figure 7A). Most of them were Class I elements such as LTR-retrotransposons [2 *Gypsy* lineages (*Tekay* and *Ogre*) and four *Copia* lineages (*SIRE, Ivana, Ikeros*, and *Ale*), *LINE* and *SINE*]. Concerning Class II elements, only *CACTA* lineages were notably expressed. *LINE* elements appear as the most highly expressed TE (Figure 7A) with an average expression level two times higher than *Gypsy Tekay* (the second highest expressed TE) and four times higher than *Copia* lineages. Expression levels of other less expressed TEs were shown in Supplementary Figure 4. Among these, mutator elements are significantly more expressed in the tetraploid *S. bakeri* than in the other tetraploid or hexaploid species.

These nine TE families were transcriptionally active in all *Spartina* species but their expression levels varied according to species (Figure 7B). The two tetraploid species showed similar and moderate TE expression levels compared to the hexaploid species. Indeed, all *Copia* lineages, as well as the *Gypsy Tekay, LINE* and *CACTA*, were significantly less expressed in *S. versicolor* and *S. bakeri* than in *S. maritima* and/or in *S. alterniflora*. Only *SINE* elements were more expressed in both tetraploids than in hexaploids (twofold more expressed in tetraploids).

Expression comparisons between the parental hexaploid species *S. maritima* and *S. alterniflora* revealed the greatest number of differentially expressed TEs (six of the nine highest expressed TEs; Figure 7B) and the greatest variation in terms of expression levels. The *Gypsy Tekay*, Ogre, the *LINEs* and the *CACTAs* elements were clearly more expressed in *S. maritima* than in *S. alterniflora* (twofold more expressed for *Gypsy Tekay*, *LINEs* and ninefold more for *CACTAs*) whereas the *Copia Ikeros* and Ale were more expressed in *S. alterniflora* than in *S. maritima* (eightfold and threefold more expressed, respectively).

In contrast, both F1 hybrids *S.* × *townsendii* and *S.* × *neyrautii* shared similar expression levels for all studied TEs. No significant differences were found except for the *Gypsy Tekay*, the *SINE* and *CACTA* elements (slightly more expressed in *S.* × *townsendii* than in *S.* × *neyrautii*). When comparing TE expression levels of the F1 hybrids with those of their parental species, statistical analyses demonstrated that most TEs were non-additively expressed in the F1 hybrids. Four of the nine studied TEs (Figure 7B) exhibited parental dominance in both or at least one F1 hybrid. The *Gypsy Tekay*, *Copia SIRE*, and *LINE* elements mimicked the expression of the maternal parent *S. alterniflora* whereas only *Gypsy Ogre* mimicked the expression of the paternal parent *S. maritima*. TEs under parental dominance in F1 hybrids displayed expressions similar to the parent with the lowest expression, especially when the parental species exhibited large expression differences. The same pattern was also observed for *SINE* elements that showed a transgressive downregulated expression in both F1 hybrids (slightly less expressed in *S.* × *townsendii* and *S.* × *neyrautii* than in the parental species).

Finally, analysis of TE expression of the allopolyploid *S. anglica* revealed that the down-regulation patterns observed in *S.* × *townsendii* were reinforced in *S. anglica*. Indeed, *Gypsy Tekay*, *Ogre*, *Copia Ivana*, *Ikeros* as well as *SINE* and *CACTA* elements were significantly less expressed in *S. anglica* than in *S.* × *townsendii*. Comparisons with expression levels detected in the parental species revealed that the expression dominance, biased toward the parent with the lowest expression level, was also strengthened in *S. anglica*: *Gypsy Tekay*, *Ogre* and *Copia Ivana* elements were found under maternal dominance in *S. anglica*, in addition to *Copia SIRE* and *LINE*s already found under the same dominance in *S.* × *townsendii*.

## Discussion

Transcriptomic changes affecting genes and transposable elements are major responses to hybridization and polyploidy. These changes that may occur immediately after (allo)polyploid speciation and persist over long-term of evolutionary time, as well as their consequences in terms of species adaptation and ecology, are timely but challenging questions. Taking advantage from the *Spartina* system where recurrent hybridization and polyploidy are well-documented in a well-understood phylogenetic context (Marchant, 1968b; Baumel et al., 2002a; Ainouche et al., 2009), we evaluated transcriptomic changes following past and recent polyploid speciation events by comparing gene and TE expressions among seven *Spartina* species with ploidy ranging from 4× to 12×. New reference transcriptomes were assembled for the tetraploid *S. bakeri* and *S. versicolor* (*syn*. *S. patens*), and the previously annotated transcriptomes were enriched for the hexaploid species (*S. alterniflora*, *S. maritima*, *S.* × *townsendii*, and *S.* × *neyrautii*) and the allododecaploid *S. anglica* (Ferreira de Carvalho et al., 2013b; Boutte et al., 2016). Ortholog detection and statistical analyses of gene and TE expression allowed comparing the expression levels of orthologous contigs included in 42,423 clusters and 21 TE lineages.

### Contrasted Gene Expression and TE Dynamics in Tetraploid and Hexaploid *Spartina* Subclades

The tetraploid lineage (2*n* = 4*x* = 40) is composed of eight *Spartina* species native to North or South America that so far have been poorly investigated regarding transcriptomic analyses (except the prairie cordgrass *Spartina pectinata*; Gedye et al., 2010; Nah et al., 2016). Here we analyzed two additional tetraploid species, *S. bakeri* and *S. versicolor*. These species show different ecological preferences (freshwater habitat and high marsh, respectively) but are weakly divergent morphologically (Mobberley, 1956) and genetically (Baumel et al., 2002a, 2016; Fortune et al., 2007; Peterson et al., 2014). Our results also show a weak transcriptome divergence as only a limited number of genes (6,111 orthologs; 14.4% of clusters) were found differentially expressed between these species (compared with other studied *Spartina*, see below). Genes differentially expressed were mainly involved in conserved metabolic processes such as cell development, transport and post-transcriptional protein modifications. However, in *S. versicolor* we observed a higher expression of genes involved in epidermis development. This result is consistent with epidermis differences identified by Maricle et al. (2009) who showed the presence of silica cells and a higher cuticle thickness in *S. versicolor* compared to *S. bakeri*. Specific analyses of genes involved in cuticle and silica cell development are necessary to confirm this causal relationship. In addition, *S. versicolor* displayed a higher expression of genes involved in abiotic stress including salt, light and cold stress response. *S. versicolor* is adapted to salt marsh conditions (Casolo et al., 2015) whereas *S. bakeri* occupies freshwater habitats. Higher tolerance to salt stress, as well as the presence of rhizomes in *S. versicolor* (absent in *S. bakeri*, Mobberley, 1956), may explain its invasiveness and its greater worldwide distribution compared to *S. bakeri*.

The majority of studied TE lineages exhibited similar expression levels in the two tetraploids. Previous investigations of repetitive sequences in the *S. bakeri* and *S. versicolor* genomes (Giraud et al., 2020) indicated that these species share the same quantity and diversity of TEs. Similar TE expression patterns thus indicate that no new significant transcriptional and/or transposition activity occurred following their divergence <2 mya, which occurred relatively recently in the *Spartina* clade (Rousseau-Gueutin et al., 2015). One exception can be made for Class II Mutator elements that appear to be ninefold more expressed in *S. bakeri* than in *S. versicolor* (Supplementary Figure 3), suggesting their recent transcriptional activation and insertion in the *S. bakeri* genome. Quantitative estimations of *Mutator* elements indicate that they are notably more abundant in *S. bakeri* (9.4 Mb) than in *S. versicolor* (1.5 Mb) genomes (Giraud et al., 2020.), which would be consistent with their reported high rates of transposition (Dupeyron et al., 2019).

In contrast to the two tetraploids investigated, the hexaploids *S. maritima* (2*n* = 6*x* = 60) and *S. alterniflora* (2*n* = 6*x* = 62) are morphologically (Mobberley, 1956) and genetically (Baumel et al., 2001, 2002a; Salmon et al., 2005; Parisod et al., 2009) well-differentiated species. They diverged about 2–4 mya along the European and American Atlantic coasts, respectively (Rousseau-Gueutin et al., 2015). About 1–5% nuclear nucleotide divergence was reported between these species (Fortune et al., 2007; Chelaifa et al., 2010a; Ferreira de Carvalho et al., 2013a, b; Boutte et al., 2016). Genome wide expression patterns are consistent with this divergence, as 15,278 differentially expressed orthologs (36.0% of studied clusters) were detected between *S. alterniflora* and *S. maritima*. Genes DE were involved in several conserved metabolic processes (cell development, protein catabolic process, glycolysis process, transport, and post-transcriptional protein modifications), but interestingly also in processes related to the physiology and ecology of *Spartina*. As previously observed using quantitative PCR analyses on target genes (Ferreira de Carvalho et al., 2017), our study confirmed that genes involved in response to salt stress were up-regulated in *S. alterniflora* compared to *S. maritima*. This result was directly in line with better tolerance to abiotic stress and invasiveness of *S. alterniflora* highlighted in several studies that investigated its leaf morphology and anatomy (Maricle et al., 2009) and its tolerance to salt and hydrocarbon stress conditions (Watts et al., 2006; Bedre et al., 2016; Cavé-Radet et al., 2019b). These results somehow differ from previous transcriptomic comparisons between *S. maritima* and *S. alterniflora* performed using rice microarrays (Chelaifa et al., 2010a), which identified only 13.3% of DE genes (1,247 among the 9,353 examined genes in both species), most of them (957, belonging to developmental and cellular growth genes) being upregulated in *S. alterniflora* and downregulated in *S. maritima*. Various causes may explain the observed differences between our results and Chelaifa et al. (2010b) findings, such as: (i) rice microarray specificity, which tends to reduce the number of analyzed genes (70% of rice genes were hybridized with *Spartina* RNA on microarray), (ii) the potentially variable expression patterns in different conditions (i.e., plants maintained in the Greenhouse in Chelaifa’s study and plants in natural conditions in this study) and different tissues (i.e., leaves and roots in Chelaifa’s study and leaves in this study).

Divergent evolution between *S. maritima* and *S. alterniflora* appears to have also resulted in significant TE expression divergence. *Gypsy Tekay*, *Ogre*, *LINEs*, and *CACTAs* elements were significantly more expressed in *S. maritima* than in *S. alterniflora*, whereas *Copia Ikeros* and *Ale* elements were more expressed in *S. alterniflora* than in *S. maritima*. The expression levels seem positively correlated with their relative abundance in both genomes. For example, *Copia Ikeros* elements, which represent 25 Mb of the *S. alterniflora* genome and 8.9 Mb of the *S. maritima* genome (Giraud et al., 2020), were eightfold more expressed in *S. alterniflora* than in *S. maritima*. These results clearly show that TE activity in *S. alterniflora* and *S. maritima* has differently evolved since their divergence 2–4 mya and probably led to some TE insertions in one or the other species according to TE lineage. Cavé-Radet et al. (2019a) identified 3,730 ra-siRNAs involved in the TE regulation in *S. maritima* and *S. alterniflora* preferentially targeting *Copia Ivana* and *SIRE*, *Gypsy Tekay* and *LINE* elements. These findings as well as our expression analyses indicate that in both hexaploids, these four active TEs are post-transcriptionally regulated (via small RNA synthesis) preventing their accumulation in the genome. However, highly expressed TEs such as *Copia Ikeros* and *Ale* in *S. alterniflora* and *CACTAs* in *S. maritima*, were not clearly under smallRNA control (Cavé-Radet et al., 2019a). This would suggest that both *Copia* elements benefit from *trans*-regulation of other expressed *Copia* elements (such as *Ivana* and *SIRE*). The limited post-transcriptional regulation of *CACTA*s despite their high transcriptional level may thus explain their accumulation in *S. maritima* (50.7 Mb vs. 26.0 Mb in *S. alterniflora*; Giraud et al., 2020).

All-to-all comparisons between tetraploid and hexaploid species revealed that the number of DE genes ranged from 27.3% (between *S. bakeri* and *S. alterniflora*) to 35.1% (between *S. versicolor* and *S. maritima*). GO-terms enrichment analyses showed that differences between tetraploid and hexaploid mainly concern genes involved in glycolysis, post-transcriptional protein modification, epidermis development. Interestingly, genes involved in the biosynthesis of carotenoids were less expressed in tetraploids than in hexaploids. Studies on plant responses to zinc and phenanthrene stresses in *Spartina densiflora* or to chromium stress in *Spartina argentinensis* (syn. *S. spartinae*; 2*n* = 4*x* = 40) exhibit a decrease of carotenoids in stress condition (Mateos-Naranjo et al., 2008; Redondo-Gomez et al., 2011; Redondo-Gómez et al., 2011). Observed variations of genes involved in the carotenoid metabolic pathway between tetraploid and hexaploid species may be due to stress conditions of salt marsh (salt stress, flooding, and pollution) or linked to functions of carotenoids in plants (antioxidant during photosynthesis, precursors for the abscisic acid synthesis). To date, it remains unclear whether transcriptomic changes observed between tetraploid and hexaploid species appeared (i) following ploidy increase, (ii) more progressively during *Spartina* evolution or (iii) both. The auto- or allo-polyploid origin of the tetraploid and hexaploid *Spartina* lineages is not yet fully elucidated, and divergence of the duplicated ancestral genomes (including regulatory elements) and their subsequent evolution must have affected the transcriptome fates of the studied species. Nuclear gene phylogenies or haplotype detection from RNA-Seq data performed so far have revealed the presence of differentiated homeologs in both tetraploid and hexaploids (Fortune et al., 2007; Boutte et al., 2016; Ferreira de Carvalho et al., 2017) which would suggest a reticulate (i.e., allopolyploid) origin of these lineages, although differentiating autopolyploidy (followed by duplicate gene divergence) from allopolyploidy in the meso-tetraploid species is a challenging task with no known related diploid species. Moreover, which ancestral tetraploid genome(s) contributed to the hexaploid ancestor of *S. maritima* and *S. alterniflora* remains an open question.

### Rapid Transcriptome Evolution Following Interspecific Hybridization: Alteration of Gene Expression and TE Silencing

The *Spartina* system offers unique opportunities to explore two components of the allopolyploid speciation process in natural conditions (hybridization versus genome duplication), a situation rarely met among recent and natural allopolyploid models (Ainouche and Wendel, 2014). Moreover, the first steps of the allopolyploid speciation process in natural conditions, i.e., consequences of divergent genome merger, can be explored in two independently formed hybrids *S.* × *townsendii* and *S.* × *neyrautii*, which share the same maternal (*S. alterniflora*) and paternal (*S. maritima*) species (Baumel et al., 2003; Ainouche et al., 2004). These two hybrids exhibit different morphologies, *S.* × *townsendii* bearing intermediate traits between the parental species, and *S.* × *neyrautii* being highly similar to *S. alterniflora* (Marchant, 1977; Baumel et al., 2003). In spite of high pollen sterility, *S.* × *townsendii* still forms vigorous populations at the hybridizing site in England (Renny-Byfield et al., 2010; Huska et al., 2016), whereas only remnant sterile *S.* × *neyrautii* individuals are surviving in south-west France as a result of site disturbance and urbanization (Hubbard, 1968; Baumel et al., 2003). The different traits of these two hybrids sharing the same genetic origin have always been puzzling. Our analyses reveal that hybridization resulted in consistent transcriptomic changes, with slightly more genes deviating from parental expression additivity in *S.* × *townsendii* (48.1%) than in *S.* × *neyrautii* (42.8%). These results confirmed the gene expression alteration following hybridization previously found in *S.* × *townsendii* and *S.* × *neyrautii* by microarray analyses (Chelaifa, 2010; Chelaifa et al., 2010b). The majority of genes considered DE compared to MPV in *S.* × *townsendii* and *S.* × *neyrautii* were found DE in both hybrids indicating consistent effects of two independent hybridization events on gene regulation evolution. More specifically, in both *S.* × *townsendii* and *S.* × *neyrautii* genes under paternal dominance were mainly involved in gene silencing processes whereas genes under maternal dominance were involved in glycolysis process, post-transcriptional protein modification and protein transport. Our results also revealed that genes involved in negative regulation and chromatin silencing were overexpressed in *S. alterniflora* compared to *S. maritima*. This negative regulation mimicking the paternal parent in hybrids, not previously reported, is consistent with DNA methylation repatterning identified following hybridization using methylation sensitive amplified polymorphism analyses (Salmon et al., 2005; Parisod et al., 2009). Results thus suggest that epigenetic modifications, which appear rapidly in the newly formed F1 hybrids, led to gene repression or silencing in *S.* × *townsendii* and *S.* × *neyrautii.* Similar transcriptomic differences were observed in rice hybrids and were shown to result from the differential production of small interfering RNA (siRNA) in the parental lines. Thus, the F1 hybrids produced siRNA at an intermediate level compared to the two parents and if the level of a siRNA produced was sufficient to methylate both homeologs, this led to the hypermethylation of the locus from the unmethylated parent and its lower transcription (Chodavarapu et al., 2012), as potentially observed in our *Spartina* hybrids.

In contrast with previous microarray analyses (Chelaifa, 2010), no significant parental expression dominance was observed for most genes in both F1 hybrids, with an equivalent number of genes under paternal and maternal dominance (16.9 vs. 16.3% in *S.* × *townsendii* and 15.2% vs. 15.1% in *S.* × *neyrautii*). However, our results agree with Chelaifa (2010) regarding genes differentially expressed between the two hybrids where over-expression of genes involved in development and cellular growth in *S.* × *neyrautii* compared to *S.* × *townsendii* was reported. Our study also found that genes transgressively up-regulated in *S.* × *neyrautii* and additive in *S.* × *townsendii* compared to MPV were mainly involved in gravitropism, development and cellular organization. These results are consistent with the known morphological differences between these hybrids: *S.* × *neyrautii* plants exhibit high stems, long and fleshy leaves and long rhizomes whereas *S.* × *townsendii* usually display small height with reduced leaves (Marchant, 1977). In agreement with the microarray study, we also found that genes involved in salt stress response were up-regulated in *S.* × *townsendii* compared to *S.* × *neyrautii*. Comparison of salt stress tolerance between these hybrids was not performed to date but could help to better understand their physiology, ecology and contrasted distribution. Finally, genes involved in gene silencing also appear more expressed in *S.* × *townsendii* than in *S.* × *neyrautii* suggesting that hybridization induced a gene expression repression more important in *S.* × *townsendii*. Interestingly, when examining the non-additive patterns (compared to the MPV) of the genes DE between the two hybrids, we surprisingly found contrasted patterns in *S.* × *townsendii* and *S.* × *neyrauti*. Thus, our results provide new insights regarding the consequences of divergent genome merger, where non-additive gene expression in independent hybridization events (involving similar parental genotypes, Baumel et al., 2003) may entail differential parental gene expression repatterning, which very well illustrates the myriads of possible outcomes resulting from the “genomic shock” of hybridization (Nieto Feliner et al., 2020). The observed expression changes could also reflect post-hybridization or post-genome duplication evolution, although this might be limited regarding the generation time in these perennial young (c.a. 150 years old) plants. Hybridization is widespread and recurrent in natural populations, which increases the hybrid population genetic diversity. Recurrent and reciprocal hybridization between diploid *Tragopogon dubius* and *T. pratensis* resulted in the formation of morphologically diverse neo-allotetraploid *T. miscellus* individuals, which exhibit consistent transcriptomic differences, departure from parental additivity and differential homeologous expression bias (Shan et al., 2020). In our case, *S.* × *neyrautii* and *S.* × *townsendii* share the same maternal parent (*S. alterniflora*, Baumel et al., 2003), in spite of morphological and transcriptomic differences, indicating different outcomes from the “replay of the evolution tape” (Gould, 1989).

Contrasted patterns are observed for repetitive sequences where TE transcriptional activity was similar in both F1 hybrids. Indeed, *Gypsy*, *Copia*, *LINEs*, *CACTAs* lineages expressed in the parental species were also highly expressed in *S.* × *townsendii* and *S.* × *neyrautii.* Both F1 hybrids exhibited similar levels and patterns of expression suggesting that both hybridization events induced similar consequences on TE activity. Comparisons with parental expression allowed classifying TEs into two main categories. On one hand, *Gypsy Ogre*, *Copia Ikeros* and *Ale* as well as *CACTA* elements were additively expressed in the F1 hybrids indicating no expression evolution following hybridization. On the other hand, *Gypsy Tekay*, *Copia SIRE*, *Ivana*, and *LINE* elements were repressed in both hybrids (mimicking the parental species displaying the lowest expression level). Small RNA analysis conducted by Cavé-Radet et al. (2019a) revealed that this second category of TEs was specially targeted by ra-siRNAs in hexaploid species. Decrease of their transcriptional activity thus seems directly assignable to small RNA regulation inherited from parental species. In addition, Parisod et al. (2009) showed, using methyl-sensitive transposon display, that following hybridization in *Spartina*, DNA methylation increased drastically near TEs. Small RNAs can also act upstream to induce DNA methylation (RdDM pathways; Axtell, 2013; Borges and Martienssen, 2015; Wendte and Schmitz, 2018) and thus reinforce TE silencing. Consequently, all these results suggest that activity of several TEs in *Spartina* hybrids was constrained by various epigenetic regulations established rapidly following hybridization, causing TE silencing and preventing putative TE burst. This is consistent with the “genomic quiescence” with no transposition burst reported for a few targeted transposable elements in *Spartina* hybrids by Baumel et al. (2002b) and Parisod et al. (2009).

### Maternal Dominance and Strengthening of TE Silencing in the Neo-Allopolyploid

In the recently formed allododecaploid *S. anglica*, we analyzed the superimposed effects of hybridization and genome duplication on one hand, and the effect of genome duplication *per se* on the other hand, by comparing expression levels of the allododecaploid *S. anglica*, with those of the hexaploid parental species *S. alterniflora* and *S. maritima* and with those of *S.* × *townsendii*. About 33.5% contigs were DE between *S.* × *townsendii* and *S. anglica*. This result demonstrates that additional gene regulation changes affected gene expression after genome doubling *per se* as previously shown in *Spartina* (Chelaifa et al., 2010b), and in other allopolyploids such as *Senecio* (Hegarty et al., 2006), *Arabidopsis* (Wang et al., 2004; Madlung et al., 2005), *Triticum* (Shaked et al., 2001) or *Tragopogon* (Buggs et al., 2011; Boatwright et al., 2018). Among the main transcriptomic changes following genome duplication, it appeared that the number of genes mimicking the maternal expression pattern (*S. alterniflora*) increased in *S. anglica* with 7,840 genes (18.5%) under maternal dominance. This is novel finding compared to previous analyses using microarrays (Chelaifa et al., 2010b) where the maternal dominance in *S.* × *townsendii* was found attenuated in *S. anglica*. Interestingly, genes involved in trichome development were under maternal dominance in *S.* × *townsendii* (mimicking *S. alterniflora*) and stayed under the same parental dominance in *S. anglica*. Several studies (e.g., in *Arabidopsis* and *Medicago sativa*; Alkio et al., 2005; Alves et al., 2017; Cavé-Radet et al., 2020) have shown the key role of trichomes in organic xenobiotics detoxification. In *Spartina*, tolerance to phenanthrene (PAH, polycyclic aromatic hydrocarbons) was increased following allopolyploidization (Cavé-Radet et al., 2019b). *S. alterniflora* was found more tolerant to phenanthrene than *S. maritima* but less tolerant than *S.* × *townsendii* and *S. anglica*. Then the trichome development regulation in the hybrid and allopolyploid, inherited from the maternal parent *S. alterniflora* may explain in part their better tolerance to PAH.

Conversely, in *S.* × *townsendii* and *S. anglica*, genes involved in silencing (chromatin silencing, histone methylation, and production of smallRNA) mimicked expression levels found in the paternal parent *S. maritima*. This result confirms that the transcriptomic repression observed following hybridization was inherited after genome doubling. This is consistent with a previous MSAP study that showed in *S. anglica* the inheritance of epigenetic marks, appearing following hybridization (Salmon et al., 2005; Parisod et al., 2009). Further comparisons between genes downregulated in *S.* × *townsendii* and *S. anglica* and miRNA-target genes identified in *Spartina* (Cavé-Radet et al., 2019a) will be interesting, to explore the link between miRNA production and decrease of such gene expression in hybrid and allopolyploid compared to parental species.

In addition to maternal dominance, an increase of transgressive genes was identified in *S. anglica* (7,434 genes in *S. anglica* vs. 6,331 in *S.* × *townsendii*), a phenomenon already observed by Chelaifa et al. (2010b). Genes transgressively expressed in the F1 hybrids and *S. anglica* (up or down-regulated compared to the parental lines) were involved in different biological processes, including epidermis development, starch metabolic process, post-transcriptional protein modifications. Among a selected set of 58 transgressively expressed genes, 28 of them showed that copies from both parental species contribute to the transgressive pattern. But in contrast, for 30 of them only one parental subgenome was involved in transgressive pattern.

Detecting homeologs in an allododecaploid species such as *Spartina anglica* is particularly challenging (Boutte et al., 2015, 2016), as the parental species are hexaploids, most likely of hybrid origins (Fortune et al., 2007) and as there is no known diploid *Spartina* that could be used as reference. The origins of the hexaploid lineage as well as the number of differentiated genomes that have been merged in the hexaploid ancestor are not fully elucidated yet. Notwithstanding this complexity and the absence of *Spartina* reference genome, we were able to take advantage from the divergence that occurred between the two hexaploid parents *S. maritima* and *S. alterniflora* in the last 2–4 my to identify polymorphic orthologous regions and detect *maritima* versus *alterniflora* haplotypes, using parental reference transcriptomes, a procedure successfully employed in recent allopolyploids [e.g., *Capsella bursa-pastoris*, Kryvokhyzha et al. (2019); *Mimulus peregrinus*, Edger et al. (2017); *Tragopogon mirus* and *T. miscellus*, Boatwright et al. (2018)]. Further analyses on the *Spartina* genomes, aiming at exploring the nature and history of monoploid genomes in tetraploids and hexaploids, will allow distinguishing the meso-homeologs in the modern polyploids.

Non-additive patterns of parental expression contribute to enhance plasticity and adaptive responses to fluctuating environments in natural allopolyploid populations (Ferreira de Carvalho et al., 2017; Shimizu-Inatsugi et al., 2017). Our results also show an over-expression of genes involved in salt stress response in *S. anglica* compared to the F1 hybrid and the parental species. Several studies reported the intercontinental invasiveness of *S. anglica* populations that cope with severe chemical or physical constraints on salt marshes and high salinity levels (An et al., 2007; Strong and Ayres, 2013; Ainouche and Gray, 2016; Wong et al., 2018). This increased tolerance in *S. anglica* could be explained by the up-regulation of genes involved in salt stress response after genome doubling. For example, haplotype detection on two up-regulated genes in *S. anglica* (compared to *S.* × *townsendii*) and specially involved in salt stress response showed two different ways of haplotype expression evolution. For the CLC-d gene encoding the chloride channel protein (Wang et al., 2015), increase of expression was induced by the up-regulation of all or only a part of parental haplotypes retrieved in *S. anglica*. However, for the gene encoding the salt-stress inducible tonoplast aquaporin 2 (Wang et al., 2014), the restoration of “*alterniflora*-type” haplotype expression may account for its up-regulation.

In addition, we observed an over-expression of genes involved in silencing in *S. anglica* compared to *S.* × *townsendii*. For example, the FACT gene is known to encode a histone chaperone that can mediate nucleosome disassembly and reassembly (Grasser, 2020). Modification of the chromatin states via such histone chaperone was shown to mediate gene expression programs and help plants to more efficiently cope with stressful conditions (Probst and Mittelsten Scheid, 2015). Thus, it is tempting to speculate that such a gene may have played a key role during the allopolyploidization process, which merged two different genomes with divergent regulatory elements in the same nucleus. The identification of the parental origin of haplotypes suggests that the up-regulation of this gene after genome doubling was due to the increase of the expression of copies from *S. alterniflora* (maternal subgenome) and silencing of “*maritima*-type” haplotypes (already observed in *S.* × *townsendii*). The upregulation of genes involved in silencing in *S. anglica* suggested not only a parental legacy of repression as indicated above but also a strengthening of repression after genome duplication.

In this paper, we compared the relative expression per transcriptomes in the hexaploid parents and F1 hybrids and the allododecaploid, by normalizing the number of reads relative to the total transcripts in each species. This assumes that mRNA transcriptome size (total number of transcripts per cell) is constant, which holds true when cells produce similar levels of RNA/cell (Lovén et al., 2012). Although widely used in comparative RNA-Seq studies, this approach does not take into account the potential absolute mRNA transcriptome size variation (reviewed Coate and Doyle, 2015) that may result from increased ploidy, which affects both gene copy number and cell size for some tissues as reported when comparing the allotetraploid *Glycine dolichocarpa* to its diploid progenitors (Coate and Doyle, 2010). These authors developed a quantitative PCR (qPCR) assay that normalizes individual gene expression to the genomic copy number when comparing the allotetraploid *G. dolichocarpa* to its diploid progenitors. Relative mRNA transcriptome sizes could be estimated by coupling this essay with transcriptome-normalized expression data (RNA-Seq) and revealed that the allotetraploid leaf transcriptome was approximately 1.4-fold larger than either diploid progenitor transcriptome. In *Spartina anglica*, gene dosage response is more complex as the parental genomes are hexaploid, and their genome composition as well as the extent of the fractionation process has to be elucidated as mentioned above. Future studies paralleling co-extracted genomic DNA and RNA Illumina sequencing should allow exploring this question. A larger sampling of additional plant organs and tissues, including root and inflorescences would be of particular interest for exploring previously reported differential tolerance to stresses (i.e., HAP and salt-stress) of investigated species and with regard to their fertility. As we are aware that transcriptome changes do not obviously correlate to the translatome (e.g., Coate et al., 2014; Soltis et al., 2016), another interesting question would be the impact of these transcriptional changes on the proteome of *S. anglica*, and its correlation to transcriptome responses.

Regarding repetitive sequences, TE repression, already observed in F1 hybrids, seems reinforced following genome doubling in *S. anglica*. Among the nine highest expressed TEs, six of them (*Gypsy Tekay*, *Ogre*, *Copia Ivana*, *Ikeros*, *SINEs*, and *CACTAs*) were less expressed in *S. anglica* than in *S.* × *townsendii*. This indicates that additional regulation takes place following genome doubling to repress TE transcriptional activity. Studies on *Arabidopsis* or *Triticum* polyploids showed that allopolyploidization induced rapid methylation changes near TEs, avoiding TE burst (Madlung et al., 2005; Parisod et al., 2010a; Ben-David et al., 2013). Analyses in *Spartina* also revealed that DNA methylation that appeared near TEs following hybridization were conserved after genome doubling (Parisod et al., 2009). Therefore, in *S. anglica*, TE transcriptional activity was partly under the control of repressive epigenetic marks already implemented following hybridization and inherited from *S.* × *townsendii*.

In conclusion, our comparative transcriptomic analyses among *Spartina* species allowed us to understand the evolution of gene and TE expression following recent and past polyploidization events. Gene expression changes were consistent with phylogenetic relationships and divergence time between species. Comparisons of tetraploid and hexaploid species showed that the TE dynamics was clearly different, reflecting a complex evolutionary history in both lineages since their divergence 6–10 mya. Particularly remarkable is the significant transcriptome repatterning following reticulate evolution, where expression changes (consistent with epigenetic and regulatory mechanisms alterations) that took place in 150–170 years old hybrids and neo-allopolyploid *S. anglica* far exceeded long term divergent transcriptome evolution in the meso-tetraploid and meso-hexaploid lineages. The superimposed polyploidization events which took place in the *Spartina* clade during the last 10–12 my offered increased opportunity to partition parental expression. Recent allopolyploidy provided springboards for new regulatory and expression patterns that played a central role in the species traits and ecology, including abilities to colonize stressful and fluctuating environments on saltmarshes, as particularly well illustrated in the worldwide invasive allododecaploid *S. anglica*. The genomic and transcriptomic resources being developed on this system now open new perspectives to explore the deepest history of the parental species, the extent of fractionation affecting the ancestral tetraploid and hexaploid genomes, and the way this dynamic affects adaptation and invasiveness of the modern species.

## Data Availability Statement

The datasets generated for this study are available at the NCBI SRA database on Bioproject PRJNA657699. Data used for reference transcriptome assemblies by Boutte et al. (2016) are available on BioProject PRJNA338100 (www.ncbi.nlm.nih.gov/sra/?term=PRJNA338100).

## Author Contributions

MA, AS, and MR-G designed the experiments. OL contributed to molecular experiments for RNA seq. DG, AS, MA, and MR-G analyzed the results and wrote the manuscript. All authors contributed to the article and approved the submitted version.

## Conflict of Interest

The authors declare that the research was conducted in the absence of any commercial or financial relationships that could be construed as a potential conflict of interest.
