# Supplementary material for: Gene and Transposable Element Expression Evolution Following Recent and Past Polyploidy Events in Spartina (Poaceae)
Source: Front Genet. 2021 Mar 25;12:589160. doi: 10.3389/fgene.2021.589160 (PMC8027259; doi:10.3389/fgene.2021.589160)
Supplement: Supplementary file 1 [file Data_Sheet_1.PDF]

Supplementary Material

Supplementary Table 1. Summary of RNA sequencing libraries used for the analyses.

Species	Individual	Replicates	Number of paired reads
S. maritima (6x)	Individual 1	Lane 3	31,626,301
		Lane 4	33,779,293
	Individual 2	Lane 1	53,827,244
		Lane 6	27,205,945
S. alterniflora (6x)	Individual 1	Lane 3	49,378,483
		Lane 4	45,693,195
	Individual 2	Lane 2	56,242,218
		Lane 6	28,478,562
S. x townsendii (6x)	Individual 1	Lane 3	40,457,540
		Lane 4	41,197,884
	Individual 2	Lane 2	58,576,032
		Lane 6	31,542,362
S. x neyrautii (6x)	Individual 1	Lane 1	55,531,378
	Individual 2	Lane 2	55,450,775
		Lane 7	28,604,363
S. anglica (12x)	Individual 1	Lane 3	34,863,249
		Lane 4	32,669,000
		Lane 5	49,372,122
		Lane 7	30,178,707
	Individual 2	Lane 1	52,562,607
		Lane 5	49,490,626
		Lane 6	24,836,930
		Lane 7	22,400,781
S. versicolor (4x)	Individual 1	Lane 6	26,817,283
		Lane 7	25,378,068
	Individual 2	Lane 7	24,770,631
S. bakeri (4x)	Individual 1	Lane 5	56,111,762
	Individual 2	Lane 7	29,727,739

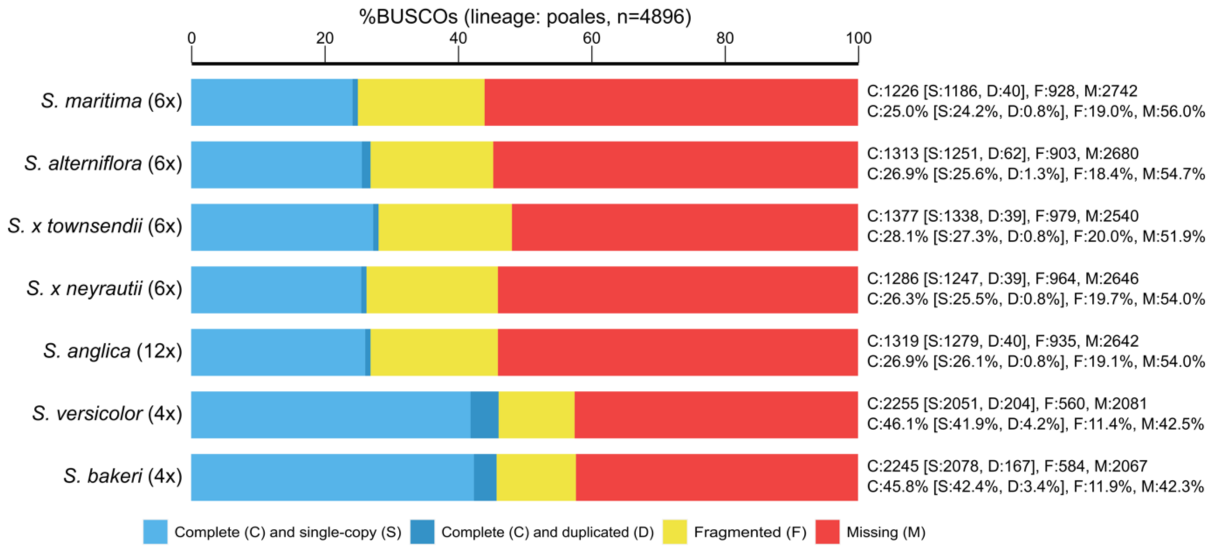

Supplementary Figure 1. BUSCO quality assessments for the seven assembled transcriptomes. Transcriptomes of the parental hexaploid species *S. maritima* and *S. alterniflora* and their derived taxa (both hybrids *S. x townsendii* and *S. x neyrautii* and the allododecaploid *S. anglica*) showed similar features: 25.0%-28.1% out of 4,896 BUSCO genes are complete, of which 24.2%-27.3% are single-copy and 0.8%-1.3% are multiple copies. Other BUSCO genes are fragmented (18.4%-20.0%) or missing (51.9%-56.0%). For tetraploid species *S. versicolor* and *S. bakeri*, we found 46.1% and 45.8% of complete BUSCO genes in their respective transcriptomes (41.9% and 42.4% are single copy; 4.2% and 3.4% are multiple copies). Other BUSCO genes are fragmented (11.4%-11.9%) or missing (42.3%-42.5%).

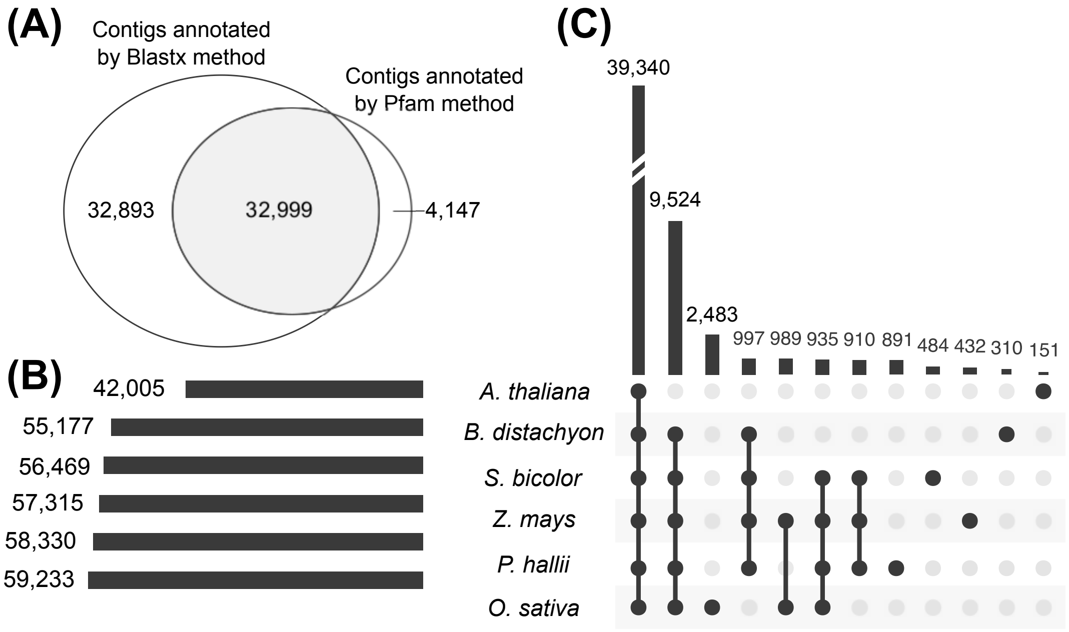

Supplementary Figure 2. Contig annotation of the *S. maritima* transcriptome with the BLASTx method (alignment on 6 protein databases from reference plant genomes: *Arabidopsis thaliana*, *Oryza sativa*, *Brachypodium distachyon*, *Panicum hallii*, *Sorghum bicolor*, *Zea mays*) and the Pfam method (alignment on the Pfam database with the hmmer program). **(A)** Venn Diagram representing the number of annotated contigs by one or both methods in *S. maritima*. **(B)** Number of annotated contigs with the BLASTx method for each reference species. **(C)** Contribution of each reference species in the contig annotation of *S. maritima* with BLASTx (*e.g.* 39,340 contigs were annotated by all reference species whereas 2,483 contigs were annotated only by *Oryza sativa* protein database).

Supplementary Table 2. Number of annotated contigs used for orthologs identification and results of OrthoVenn2 clustering

**Species**	***S. maritima***	***S. alterniflora***	***S. x townsendii***	***S. x neyrautii***	***S. anglica***	***S. versicolor***	***S. bakeri***
**Number of annotated contigs**	76,916	69,980	93,645	91,798	94,130	66,924 (65%)	62,827
**Percentage of mapped reads on annotated contigs (mean of lanes)**	85%	83%	83%	83%	83%	92%	92%
**Number of contigs included in clusters of orthologs**	50,245 (65%)	45,940 (66%)	59,264 (63%)	58,605 (64%)	55,933 (59%)	41,832 (62%)	39,817 (63%)
**Number of clusters containing at least one contig of the species**	34,952 (56%)	31,544 (50%)	41,993 (67%)	41,280 (66%)	38,652 (62%)	25,969 (40%)	24,987 (41%)

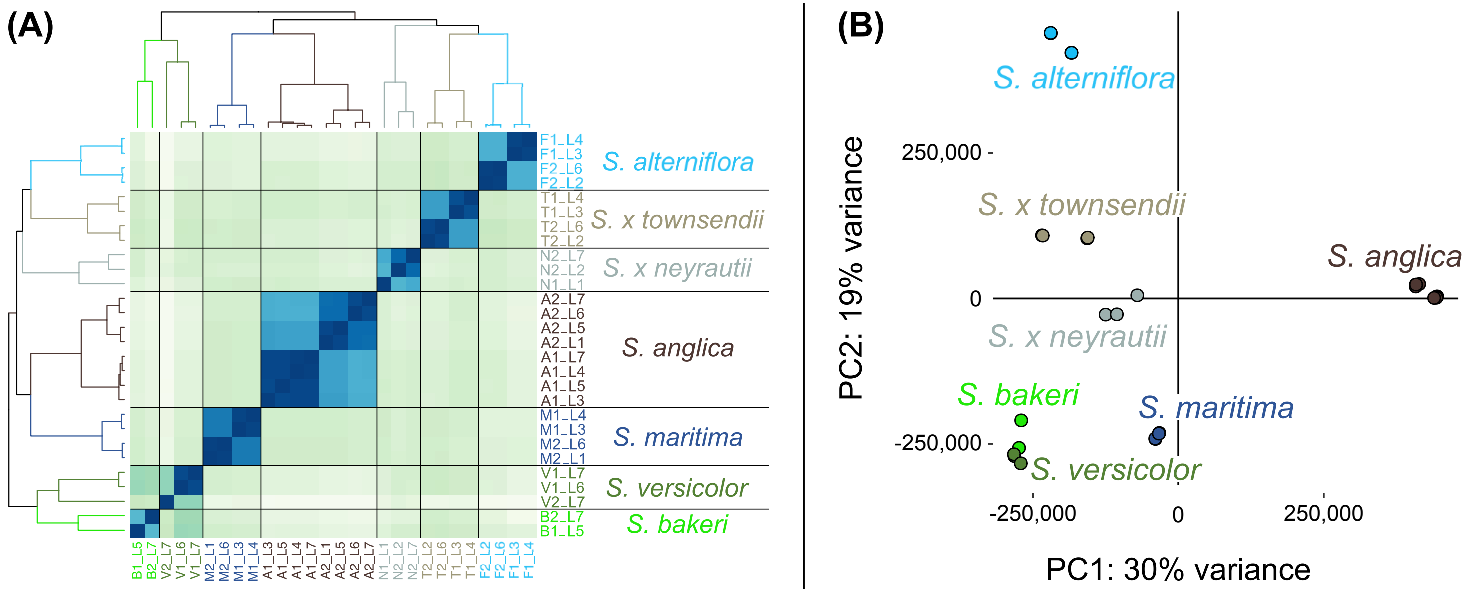

Supplementary Figure 3. Assessment of data quality and reproducibility based on sample clustering via Euclidean distances **(A)** and principal component analysis. **(B)** After normalization, gene counts obtained for each library were compared to detect potential sample errors.

Supplementary Table 4. Biological functions (list of enriched GO-terms) of genes differentially expressed between tetraploids (*S. versicolor* and *S. bakeri)* or between hexaploid (*S. alterniflora* and *S. maritima)*. GO-terms are significantly enriched when adjusted p-value (in brackets) is less than 0.01. Number of contigs corresponds to the number of genes DE between species.

S. versicolor > S. bakeri	**Nb contigs: 2,933**
Epidermis development	GO:0009793 (2.44e-05) embryonic development ending in seed dormancy
	GO:0048767 (0.000648) root hair elongation
	GO:0010090 (0.00173) trichome morphogenesis
	GO:0048764 (0.00026) trichoblast maturation
	GO:0008544 (4.54e-06) epidermis development
	GO:0009640 (0.00238) photomorphogenesis
Proteasome assembly	GO:0043248 (0.00061) proteasome assembly
	GO:0080129 (0.00226) proteasome core complex assembly
Response to biotic/abiotic stress	GO:0009651 (4.18e-10) response to salt stress
	GO:0046686 (1.24e-16) response to cadmium ion
	GO:0051788 (0.000741) response to misfolded protein
	GO:0006974 (0.001) response to DNA damage stimulus
	GO:0042742 (0.00751) defense response to bacterium
	GO:0009416 (8.01e-11) response to light stimulus
	GO:0009409 (1.1e-06) response to cold
Glycolysis process	GO:0006096 (1.57e-10) glycolysis
	GO:0006007 (1.31e-12) glucose catabolic process
	GO:0006094 (2.67e-10) gluconeogenesis
Gene silencing	GO:0045892 (0.000147) negative regulation of transcription, DNA-dependent
	GO:0006342 (0.000448) chromatin silencing
	GO:0031047 (0.00946) gene silencing by RNA
	GO:0045814 (0.000407) negative regulation of gene expression, epigenetic
Nucleotide biosynthetic	GO:0009220 (0.00374) pyrimidine ribonucleotide biosynthetic process
	GO:0009165 (8.09e-06) nucleotide biosynthetic process
Post-transcriptional protein modification	GO:0019941 (1.27e-05) modification-dependent protein catabolic process
	GO:0006511 (1.42e-05) ubiquitin-dependent protein catabolic process
	GO:0010498 (3.79e-06) proteasomal protein catabolic process
	GO:0018319 (0.000832) protein amino acid myristoylation
	GO:0006498 (0.000832) N-terminal protein lipidation
	GO:0006499 (0.000832) N-terminal protein myristoylation

S. versicolor < S. bakeri	**Nb contigs: 3.178**
Cell development	GO:0022403 (1.3e-05) cell cycle phase
	GO:0000279 (0.000747) M phase
	GO:0016568 (0.0029) chromatin modification
	GO:0007062 (3.34e-05) sister chromatid cohesion
Protein modification	GO:0006468 (9.33e-10) protein amino acid phosphorylation
	GO:0006487 (6.38e-08) protein amino acid N-linked glycosylation
Protein transport	GO:0015031 (4.86e-07) protein transport
	GO:0006886 (7.68e-07) intracellular protein transport
	GO:0006605 (7.24e-06) protein targeting

S. alterniflora > S. maritima	**Nb contigs: 7,482**
Response to abiotic stress	GO:0009651 (9.93e-10) response to salt stress
	GO:0009409 (1.09e-06) response to cold
Cytoskeleton organization	GO:0007010 (3.74e-11) cytoskeleton organization
	GO:0007015 (0.00196) actin filament organization
	GO:0045010 (0.00301) actin nucleation
Cell development	GO:0022403 (3.39e-06) cell cycle phase
	GO:0000279 (3.93e-06) M phase
	GO:0016049 (2.75e-06) cell growth
Fatty acid metabolic process	GO:0019752 (1.7e-20) carboxylic acid metabolic process
	GO:0030258 (4.1e-09) lipid modification
	GO:0006631 (9.97e-09) fatty acid metabolic process
	GO:0006635 (3.04e-06) fatty acid beta-oxidation
	GO:0006633 (0.0008) fatty acid biosynthetic process
NADP metabolic process	GO:0006739 (0.00102) NADP metabolic process
	GO:0006740 (0.00266) NADPH regeneration
	GO:0006098 (0.00234) pentose-phosphate shunt
Starch biosynthetic process	GO:0009250 (8.07e-08) glucan biosynthetic process
	GO:0019252 (0.00546) starch biosynthetic process
Post-translational protein modification	GO:0043687 (9.85e-22) post-translational protein modification
	GO:0016926 (0.00108) protein desumoylation
	GO:0006498 (0.00425) N-terminal protein
	GO:0018319 (0.00425) protein amino acid myristoylation
	GO:0006499 (0.00425) N-terminal protein myristoylation
Gene silencing	GO:0051253 (0.000571) negative regulation of RNA metabolic process
	GO:0045892 (0.000571) negative regulation of transcription, DNA-dependent
	GO:0006342 (4.41e-05) chromatin silencing
	GO:0045814 (4.35e-05) negative regulation of gene expression, epigenetic

S. alterniflora < S. maritima	**Nb contigs: 7.805**
Protein catabolic process	GO:0051603 (1.15e-08) proteolysis involved in cellular protein catabolic process
	GO:0010498 (0.000721) proteasomal protein catabolic process
	GO:0019941 (1.29e-05) modification-dependent protein catabolic process
	GO:0006511 (1.3e-05) ubiquitin-dependent protein catabolic process
Post-translational protein modification	GO:0043687 (1.12e-19) post-translational protein modification
	GO:0006468 (6.62e-06) protein amino acid phosphorylation
	GO:0006479 (0.000204) protein amino acid methylation
	GO:0016571 (0.000126) histone methylation
	GO:0016569 (9.05e-06) covalent chromatin modification
Carbohydrate catabolic process	GO:0044275 (2.14e-11) cellular carbohydrate catabolic process
	GO:0034637 (2.32e-07) cellular carbohydrate biosynthetic process
Glycolysis process	GO:0006096 (2.14e-07) glycolysis
	GO:0006094 (0.000321) gluconeogenesis
	GO:0006007 (6.56e-10) glucose catabolic process
	GO:0006090 (1.02e-08) pyruvate metabolic process
Protein transport	GO:0006886 (1.46e-15) intracellular protein transport
	GO:0006605 (1.93e-11) protein targeting
Cell development	GO:0016049 (0.00652) cell growth
	GO:0009658 (3.96e-06) chloroplast organization
	GO:0010027 (0.000384) thylakoid membrane organization
Epidermis developement	GO:0009965 (0.0021) leaf morphogenesis
	GO:0035315 (0.00868) hair cell differentiation
	GO:0010026 (0.00868) trichome differentiation
	GO:0009793 (1.21e-09) embryonic development ending in seed dormancy

Supplementary Table 5. List of enriched GO-terms (biological process) when analysing DE genes between one tetraploid and one hexaploid species. GO-terms are significantly enriched when adjusted p-value (given in table) is lower than 0.01.

GO term	*S. bakeri* vs. *S. alterniflora*	*S. bakeri* vs. *S. maritima*	*S. bakeri* vs. *S. x townsendii*	*S. bakeri* vs. *S. x neyrautii*	*S. bakeri* vs. *S. anglica*	*S. versicolor* vs. *S. alterniflora*	*S. versicolor* vs. *S. maritima*	*S. versicolor* vs. *S. x townsendii*	*S. versicolor* vs. *S. x neyrautii*	*S. versicolor* vs. *S. anglica*
**Nucleotide metabolic process**										
GO:0009218 pyrimidine ribonucleotide metabolic process	0.0025	0.0073	0.006		0.0088		0.0042	0.00022	0.0015	0.0006
GO:0006221 pyrimidine nucleotide biosynthetic process	0.0023	0.007	0.0057		0.0086		0.0072	0.00023	0.0015	0.00037
GO:0009150 purine ribonucleotide metabolic process		0.0047		0.0023			0.0011	0.0045	0.0066	
GO:0009260 ribonucleotide biosynthetic process	5.5e-05	9.2e-06	4.8e-05	1.4e-05	4.4e-06		6.6e-06	1,00E-07	3.9e-07	4.5e-06
GO:0009220 pyrimidine ribonucleotide biosynthetic process	0.0082		0.01				0.01	0.0003	0.0023	0.0013
**Post-transcriptional protein modification**										
GO:0006499 N-terminal protein myristoylation	0.00015			0.00055		0.00013			0.00036	0.0082
GO:0006498 N-terminal protein lipidation	0.00015			0.00055		0.00013			0.00036	0.0082
GO:0018409 peptide or protein amino-terminal blocking	0.00015			0.00055		0.00013			0.00036	0.0082
GO:0043161 proteasomal ubiquitin-dependent protein catabolic process		9.4e-05	0.0069		0.0025	0.0054	0.0031			0.0032
GO:0016567 protein ubiquitination	0.0039	0.0043		0.0035			0.0092	0.0021		0.0014
GO:0018319 protein amino acid myristoylation	0.00015			0.00055		0.00013			0.00036	0.0082
GO:0046777 protein amino acid autophosphorylation	0.022		0.009				0.0012	0.001	2.3e-06	0.0043
GO:0006511 ubiquitin-dependent protein catabolic process	4.3e-10	1.5e-09	1.6e-08	1.5e-07	8.2e-09	1.4e-07	2,00E-07	3.6e-06	5.9e-07	2.7e-07
GO:0019252 starch biosynthetic process	2.5e-06	0.00057	1.1e-05	7.5e-05	0.13	1.2e-06	0.00048	1.3e-07	9.5e-09	0.00057
GO:0016926 protein desumoylation								0.002		0.0048
GO:0010498 proteasomal protein catabolic process	8.2e-07	1.6e-08	2.8e-08	4.9e-08	8.6e-08	2.1e-07	1.3e-06	3.3e-06	4.8e-07	1.4e-06
**Sulfur synthetic process**										
GO:0000097 sulfur amino acid biosynthetic process		4.4e-05	0.00023	0.0028		0.00048	0.00015	7.9e-05	0.00084	
**Pigment biosynthetic process**										
GO:0016117 carotenoid biosynthetic process	0.0021	0.00056	3.2e-06	1.2e-05		0.00016	0.00051	1.6e-05	7.9e-07	0.00012
GO:0016109 tetraterpenoid biosynthetic process	0.0021	0.00056	3.2e-06	1.2e-05		0.00016	0.00051	1.6e-05	7.9e-07	0.00012
GO:0016116 carotenoid metabolic process	0.0031	0.00088	1.2e-05	4.3e-05		7,00E-05	0.00047	3,00E-05	7.2e-07	0.00023
**Epidermis / trichome development**										
GO:0010026 trichome differentiation		0.0017		4.3e-05	0.0014	0.00032	9.9e-05	8.5e-05	0.0023	0.00017
GO:0010054 trichoblast differentiation				0.0028			0.0023	0.00056	0.0041	0.00066
GO:0009965 leaf morphogenesis	0.00069	1.4e-06	1.8e-05	2.8e-07	7.1e-05	3.5e-06	4.3e-06	1.9e-07	0.00015	1.3e-05
GO:0009913 epidermal cell differentiation	0.0064		0.0025	5.3e-06	0.00035	0.001	6.6e-06	5,00E-06	4.2e-05	1.8e-05
GO:0048764 trichoblast maturation	0.0039			0.00024	0.002	0.001	0.00013	2.7e-05	0.00047	4.4e-05
GO:0010090 trichome morphogenesis	0.005	0.00082		1.9e-05	0.0011	0.00012	9.9e-05	1.4e-05	0.00056	2.6e-05
GO:0010053 root epidermal cell differentiation				0.00071		0.0061	0.00054	0.00029	0.00084	0.00038
GO:0035315 hair cell differentiation		0.0017		4.3e-05	0.0014	0.00032	9.9e-05	8.5e-05	0.0023	0.00017
GO:0048765 root hair cell differentiation	0.0039			0.00024	0.002	0.001	0.00013	2.7e-05	0.00047	4.4e-05
**Fatty acid process**										
GO:0006635 fatty acid beta-oxidation	1.5e-07	4.9e-05	0.00068	0.00047	4,00E-08	9,00E-06	0.0001	0.0008	0.0038	4.8e-08
GO:0009062 fatty acid catabolic process	2.2e-05	0.0014			4.6e-06	0.00045	0.0046	0.0098		1.3e-06
GO:0019395 fatty acid oxidation	4.4e-07	0.00013	0.0016	0.0011	1.3e-07	2.5e-05	0.00027	0.0019	0.0087	1.6e-07
GO:0006633 fatty acid biosynthetic process	0.0034		0.0027	0.0014	0.00065	0.00053				0.0077
**Negative regulation / gene silencing**										
GO:0016571 histone methylation	0.0059	0.00021	0.00071	0.0023	0.0087	0.003		0.0025		0.0031
GO:0035196 production of miRNAs involved in gene silencing by miRNA			0.0016					0.0007		1.3e-05
GO:0031047 gene silencing by RNA		0.0021	0.0014			0.00023	0.0097	0.008		0.00016
GO:0006342 chromatin silencing	8.2e-06	3,00E-06	2.6e-06	6.8e-05	0.00012	6.7e-07	0.00028	5.3e-05	0.00081	2.7e-07
GO:0010267 production of ta-siRNAs involved in RNA interference								0.0049		0.00012
GO:0030422 production of siRNA involved in RNA interference			0.00092			0.009		0.00096		2.3e-05
GO:0016246 RNA interference		0.0047	0.0097			0.00062		0.0016		5.4e-05
GO:0006479 protein amino acid methylation		0.00045	0.0009	0.0028		0.004		0.0053		0.0046
GO:0045892 negative regulation of transcription, DNA-dependent	2.3e-06	7.2e-06	5.6e-07	5.2e-06	6.6e-06	3.7e-06	0.00013	6.7e-05	0.00066	2.3e-07
GO:0070918 production of small RNA involved in gene silencing by RNA		0.0076	0.00058					0.0021		3.1e-05
**Glycolysis process**										
GO:0006096 glycolysis	7.2e-12	1.1e-11	1.1e-14	3.3e-15	3.8e-14	3.2e-11	6.9e-10	5.3e-10	6.8e-14	1.5e-10
GO:0006091 generation of precursor metabolites and energy	3.1e-15	9.6e-16	2.7e-19	5.6e-20	1.1e-14	9.2e-17	5.3e-17	7.9e-21	7.2e-22	3.4e-16
GO:0006094 gluconeogenesis	2.2e-08	1.4e-06	1.6e-10	1.6e-10	2.5e-08	2,00E-09	6.5e-06	8.6e-07	1.3e-09	6.6e-07
GO:0006769 nicotinamide metabolic process	8.2e-07	2.5e-05	5.4e-08	8.2e-10	0.00047	1.7e-08	2.3e-06	5.5e-08	6.9e-12	2,00E-05
GO:0006739 NADP metabolic process	1.2e-06	0.00018	2.7e-07	2.2e-09	0.0017	7.2e-08	4.6e-06	2,00E-07	1.5e-11	0.0001
GO:0006740 NADPH regeneration	2.5e-06	0.00034	5.2e-07	9.2e-09	0.0029	1.3e-07	1.4e-05	3.5e-07	5.8e-11	0.00017
GO:0006007 glucose catabolic process	2,00E-21	9,00E-19	1.1e-22	4.1e-25	1.2e-20	8.8e-22	4.1e-18	2.3e-19	3.5e-27	5.1e-18
GO:0019288 isopentenyl diphosphate biosynthetic process, mevalonate-i	9.5e-06	9.2e-06	2.3e-11	4.2e-09	0.00054	1,00E-06	7.2e-08	6.4e-11	5.3e-09	0.001
GO:0006098 pentose-phosphate shunt	2,00E-06	0.00049	4.2e-07	1.5e-08	0.0025	1.1e-07	1.1e-05	2.8e-07	9.1e-11	0.00014
GO:0046496 nicotinamide nucleotide metabolic process	8.2e-07	2.5e-05	5.4e-08	8.2e-10	0.00047	1.7e-08	2.3e-06	5.5e-08	6.9e-12	2,00E-05
**Calcium ion transport**										
GO:0006816 calcium ion transport	0.00021		0.0018	0.0057	0.007				0.00029	0.0023

Supplementary Table 6. List of enriched GO-terms (biological process) when analysing DE genes following hybridization and/or genome doubling. GO-terms are significantly enriched when adjusted p-value (given in table) is lower than 0.01.

GO term	*S. alterniflora* vs. *S. maritima*	*S. x townsendii* vs. MPV	*S. x townsendii* vs. *S. alterniflora*	*S. x townsendii* vs. *S. maritima*	*S. x neyrautii* vs. MPV	*S. x neyrautii* vs. *S. alterniflora*	*S. x neyrautii* vs. *S. maritima*	*S. anglica* vs. MPV	*S. anglica* vs. *S. alterniflora*	*S. anglica* vs. *S. maritima*	*S. x neyrautii* vs. *S. x townsendii*	*S. anglica* vs. *S. x townsendii*
**Nucleotide metabolic process**												
GO:0009205 purine ribonucleoside triphosphate metabolic process				0.0098			5,00E-05				0.00021	
GO:0009218 pyrimidine ribonucleotide metabolic process		0.0008		0.00033			0.00053	7.6e-06	0.0051	3.7e-07		
GO:0009144 purine nucleoside triphosphate metabolic process				0.0098			5,00E-05				0.00021	
GO:0006221 pyrimidine nucleotide biosynthetic process		0.0015		0.00058			0.0009	5.2e-06	0.005	2,00E-07		
GO:0006468 protein amino acid phosphorylation	4.2e-09	1,00E-08	3.4e-06	1.1e-10	1.5e-12	1,00E-09	1.7e-09	6.7e-11	1.3e-10	5.8e-12	3.5e-11	4.6e-15
GO:0009199 ribonucleoside triphosphate metabolic process				0.0098			5,00E-05				0.00021	
GO:0009150 purine ribonucleotide metabolic process				0.0058			2.4e-05	0.0099		0.0068	0.0016	0.0064
GO:0009260 ribonucleotide biosynthetic process	0.00059	4.2e-06	6.6e-05	6.8e-07			1.2e-08	4.6e-08	6.1e-06	8.3e-09		0.0098
GO:0009220 pyrimidine ribonucleotide biosynthetic process		0.00092		0.00055			0.0011	7.2e-06	0.0043	4.6e-07		
**Nucleic acid / protein transport**												
GO:0051028 mRNA transport		0.0017	0.0035									
GO:0006406 mRNA export from nucleus		0.0017	0.0035									
GO:0006405 RNA export from nucleus		0.0019	0.0022									
GO:0006606 protein import into nucleus		0.0029	0.0075									
GO:0006612 protein targeting to membrane					1.7e-05			0.0078		0.0055		
GO:0016558 protein import into peroxisome matrix		0.002			0.0083	0.0085		0.00096	0.00074		0.0051	0.0093
**Post-transcriptional protein modification**												
GO:0006499 N-terminal protein myristoylation			0.0027		0.0033	0.0039	0.0074	0.0046	0.00061			0.00022
GO:0006498 N-terminal protein lipidation			0.0027		0.0033	0.0039	0.0074	0.0046	0.00061			0.00022
GO:0018409 peptide or protein amino-terminal blocking			0.0027		0.0033	0.0039	0.0074	0.0046	0.00061			0.00022
GO:0043161 proteasomal ubiquitin-dependent protein catabolic process	0.0098	0.0031		0.00066	0.0022	0.00044	0.0032	0.0043	0.0013		0.00098	
GO:0016567 protein ubiquitination	0.00078	2.8e-06	3.4e-05	4,00E-06		0.0033	0.00013	0.0012	0.0082	0.003	0.0015	0.0071
GO:0018319 protein amino acid myristoylation			0.0027		0.0033	0.0039	0.0074	0.0046	0.00061			0.00022
GO:0000338 protein deneddylation	0.0048		0.0045	0.0035			0.0082	0.0032		0.0017		
GO:0046777 protein amino acid autophosphorylation	0.0055			0.00093	0.0018		6.7e-06	0.0013	0.0024	0.00052		0.0005
GO:0006511 ubiquitin-dependent protein catabolic process	9.3e-06	4.7e-07	2.1e-09	5.4e-07	9.5e-07	3.1e-06	3.4e-07	2.5e-06	6.9e-09	1.8e-05	1.9e-07	4.2e-07
GO:0019252 starch biosynthetic process		0.0012	5.4e-05	0.0058			0.00093	0.0058	0.00023		0.00019	0.0022
GO:0016926 protein desumoylation	0.0017	0.0076		0.00042	0.0091	0.00015	0.0035		0.0028		9,00E-05	0.0062
GO:0010498 proteasomal protein catabolic process	6.3e-06	6.3e-07	6.2e-06	2.3e-08	1.3e-06	1.1e-07	6.1e-09	3.3e-07	1.2e-07	1.4e-08	1.2e-09	2.3e-07
GO:0010388 cullin deneddylation	0.0048		0.0045	0.0035			0.0082	0.0032		0.0017		
**Epidermis / trichome development**												
GO:0010026 trichome differentiation	0.00022	1.2e-05	1.5e-05	1.8e-05	0.00039	2.7e-07	0.00024	0.0013		0.0012	3.4e-06	3.9e-05
GO:0010054 trichoblast differentiation		0.002	8,00E-05	0.0005	0.0005	0.00011	4.2e-06			0.0011	0.00012	4.8e-05
GO:0009965 leaf morphogenesis	3.1e-05	1.2e-07	1,00E-06	2,00E-05	0.0012	3.1e-07	4.3e-06	5.1e-06	7.6e-05	7.1e-05	1.2e-06	1.3e-07
GO:0009913 epidermal cell differentiation	0.00011	1.3e-05	3.5e-06	2.8e-06	3.2e-05	1.5e-07	5.9e-07	0.0032	0.0084	3.3e-05	4.9e-08	3.9e-06
GO:0048764 trichoblast maturation	0.0011	0.00013	4.4e-06	5.7e-05	3,00E-05	4.8e-06	2.4e-07	0.0039	0.0065	6,00E-05	4,00E-05	4.2e-06
GO:0010090 trichome morphogenesis	0.00014	7.5e-06	2.2e-05	3.8e-06	0.00029	5.4e-07	3.8e-05	0.00048		0.00045	4.9e-07	8.1e-06
GO:0010053 root epidermal cell differentiation	0.0072	0.00062	5.1e-05	7.8e-05	0.00024	5.8e-05	1.3e-06	0.035		0.00038	1.8e-05	1.2e-05
GO:0035315 hair cell differentiation	0.00022	1.2e-05	1.5e-05	1.8e-05	0.00039	2.7e-07	0.00024	0.0013		0.0012	3.4e-06	3.9e-05
GO:0048765 root hair cell differentiation	0.0011	0.00013	4.4e-06	5.7e-05	3,00E-05	4.8e-06	2.4e-07	0.0039	0.0065	6,00E-05	4,00E-05	4.2e-06
**Sulfur synthetic process**												
GO:0000097 sulfur amino acid biosynthetic process	0.0046	6.5e-05	0.00073	0.00074	0.0038	0.007	0.0064	0.00014	0.0049	0.00065	0.0012	0.00068
**Fatty acid process**												
GO:0006635 fatty acid beta-oxidation	1.2e-05	4.6e-07	2.7e-05	0.00039	1.1e-05	1.2e-07	0.00011	5.7e-06	1.3e-06	0.00042	1.4e-06	5.6e-06
GO:0009062 fatty acid catabolic process	0.00014	2.5e-05	0.00071	0.0038	0.00075	6.8e-06	0.0013	3.4e-05	3.5e-06	0.0022	7.3e-06	0.00012
GO:0019395 fatty acid oxidation	3.3e-05	1.5e-06	6.9e-05	0.00092	2.9e-05	3.5e-07	0.00027	1.8e-05	3.8e-06	0.0011	3.5e-06	1.5e-05
GO:0006633 fatty acid biosynthetic process	0.0033			0.0014	0.00069			0.0013			0.00034	0.00019
**Negative regulation / gene silencing**												
GO:0016571 histone methylation	2,00E-05	3.2e-06	6.7e-07					4.1e-05		0.00063		
GO:0035196 production of miRNAs involved in gene silencing by miRNA			0.00071	0.00052								
GO:0031047 gene silencing by RNA	0.0075	0.003	1.1e-05	0.0081				0.0045				
GO:0006342 chromatin silencing	1.8e-05	3,00E-05	6.1e-07	0.0015	0.00029	0.00013	0.00062	3.3e-05	5.8e-05	0.00013	0.0021	0.0032
GO:0010267 production of ta-siRNAs involved in RNA interference			0.0095	0.0073								
GO:0030422 production of siRNA involved in RNA interference		0.0044	0.00041	0.00029	0.0024			0.0043				
GO:0034968 histone lysine methylation	0.0074	0.0013	0.00057					0.0029				
GO:0016246 RNA interference		0.0038	0.00016	0.00036	0.0086	0.0035		0.0041			0.0055	
GO:0006479 protein amino acid methylation	4.9e-05	3.7e-06	1.6e-06					0.00012		0.0009		
GO:0045892 negative regulation of transcription, DNA-dependent	8.7e-06	1.1e-05	7.3e-06	0.00024	0.00049	6.9e-05	0.00063	4.2e-06	0.0013	1.1e-05	0.0004	0.00065
GO:0070918 production of small RNA involved in gene silencing by RNA		0.0035	0.00025	0.00034	0.0047			0.006			0.0076	
**Glycolysis process**												
GO:0006096 glycolysis	2.4e-10	4.7e-12	3,00E-12	4.7e-10	5.7e-15	6.1e-16	7.9e-11	1.5e-10	9.5e-12	2.3e-12	7.6e-13	6.3e-15
GO:0006091 generation of precursor metabolites and energy	3.3e-10	8.5e-14	3.5e-12	1.2e-10	5,00E-12	7.7e-12	5,00E-10	3.2e-17	2.5e-11	9.4e-17	5.9e-11	9.3e-17
GO:0006094 gluconeogenesis	1.2e-06	1.6e-07	1.7e-07	4.2e-07	2,00E-07	7.7e-08	7.2e-09	3.9e-08	3.1e-08	1.2e-10	3.5e-09	8.6e-11
GO:0006769 nicotinamide metabolic process	3.6e-06	0.00072	0.0048			0.00013	0.00012	3,00E-05	0.00012		0.00037	0.00028
GO:0006739 NADP metabolic process	2.4e-05	0.0018				0.0004	0.00021	0.00011	0.00043		0.00073	0.00069
GO:0006740 NADPH regeneration	7.2e-05	0.0027				0.0014	0.00076	0.00025	0.00074		0.0029	0.0013
GO:0006007 glucose catabolic process	1.1e-17	4.1e-17	1.2e-15	1.5e-11	2.8e-17	9.8e-20	1.8e-15	1.2e-16	4.2e-18	5.9e-15	4,00E-15	3.5e-20
GO:0019288 isopentenyl diphosphate biosynthetic process, mevalonate-i	5.2e-06	0.00052	0.00057			1.7e-05	0.0026	0.00068		0.00082	0.0012	9.6e-07
GO:0006098 pentose-phosphate shunt	6,00E-05	0.0023				0.0022	0.0012	0.00021	0.00063	0.047	0.0046	0.0011
GO:0046496 nicotinamide nucleotide metabolic process	3.6e-06	0.00072	0.0048			0.00013	0.00012	3,00E-05	0.00012	0.017	0.00037	0.00028
**Calcium ion transport**												
GO:0006816 calcium ion transport	0.0081	0.0035	0.0001	0.0003	0.00013	4.9e-05		0.0003	0.00015	0.0019	1.1e-05	0.00027

Supplementary Table 7. Biological functions (list of enriched GO-terms) of differentially expressed genes between the F1 hybrids *S. x townsendii* and *S. x neyrautii*. Genes were classified according to the hybrid expression pattern (additive, transgressive, under parental dominance). GO-terms are significantly enriched when adjusted p-value (in brackets) is lower than 0.01. Number of contigs corresponds to the number of DE genes between hybrids and that followed the indicated pattern (percentage among 10,703 DE contigs).

Pattern in *S. x townsendii*	under maternal dominance	Pattern in *S. x neyrautii*	under paternal dominance	Nb contigs: 1,639 (15.3%)
GO:0006468 (1.73e-06) protein amino acid phosphorylation				
GO:0030243 (0.00122) cellulose metabolic process				
GO:0006096 (4.73e-05) glycolysis				
GO:0016310 (3.71e-06) phosphorylation				
GO:0009651 (6.77e-12) response to salt stress				
GO:0009409 (0.000224) response to cold				
GO:0009737 (5.78e-07) response to abscisic acid stimulus				
GO:0046686 (0.000212) response to cadmium ion				
GO:0007242 (8.85e-05) intracellular signaling cascade				
GO:0016049 (0.00283) cell growth				
GO:0010090 (0.005) trichome morphogenesis				
GO:0006816 (0.000285) calcium ion transport				
GO:0006888 (0.000965) ER to Golgi vesicle-mediated transport				
GO:0006886 (0.00124) intracellular protein transport				

Pattern in *S. x townsendii*	under paternal dominance	Pattern in *S. x neyrautii*	under maternal dominance	Nb contigs: 1,682 (15.7%)
GO:0009793 (0.00267) embryonic development ending in seed dormancy				
GO:0006007 (0.00449) glucose catabolic process				
GO:0006511 (5.97e-05) ubiquitin-dependent protein catabolic process				
GO:0006468 (1.96e-07) protein amino acid phosphorylation				
GO:0046686 (1.22e-05) response to cadmium ion				
GO:0048765 (0.0065) root hair cell differentiation				
GO:0048764 (0.0065) trichoblast maturation				
GO:0016049 (0.000301) cell growth				
GO:0009416 (0.00619) response to light stimulus				
GO:0009651 (0.00863) response to salt stress				
GO:0045087 (0.00549) innate immune response				

Pattern in *S. x townsendii*	transgressive down-regulated	Pattern in *S. x neyrautii*	additive	Nb contigs: 784 (7.3%)
GO:0006950 (6.94e-05) response to stress				
GO:0034637 (0.00146) cellular carbohydrate biosynthetic process				
GO:0006396 (3.09e-05) RNA processing				
GO:0051603 (0.000228) proteolysis involved in cellular protein catabolic process				

Pattern in *S. x townsendii*	additive	Pattern in *S. x neyrautii*	transgressive down-regulated	Nb contigs: 539 (5.0%)
GO:0044262 (9.08e-06) cellular carbohydrate metabolic process				
GO:0009853 (0.00758) photorespiration				
GO:0009628 (0.00341) response to abiotic stimulus				
GO:0044260 (0.00104) cellular macromolecule metabolic process				

Pattern in *S. x townsendii*	transgressive up-regulated	Pattern in *S. x neyrautii*	additive	Nb contigs: 1,378 (12.9%)
GO:0010228 (0.000474) vegetative to reproductive phase transition of meristem				
GO:0010054 (0.00988) trichoblast differentiation				
GO:0045087 (1.01e-06) innate immune response				
GO:0009651 (1.77e-08) response to salt stress				
GO:0046686 (0.000408) response to cadmium ion				
GO:0051607 (0.00236) defense response to virus				
GO:0009627 (0.000999) systemic acquired resistance				
GO:0042742 (0.000724) defense response to bacterium				
GO:0009737 (0.00919) response to abscisic acid stimulus				
GO:0009755 (0.00446) hormone-mediated signaling pathway				
GO:0031050 (0.00398) dsRNA fragmentation				
GO:0070918 (0.00398) production of small RNA involved in gene silencing by RNA				
GO:0030422 (0.00286) production of siRNA involved in RNA interference				
GO:0006468 (0.00311) protein amino acid phosphorylation				
GO:0005982 (0.00248) starch metabolic process				
GO:0006007 (0.00132) glucose catabolic process				
GO:0032787 (6.32e-09) monocarboxylic acid metabolic process				
GO:0006886 (0.00118) intracellular protein transport				
GO:0006816 (0.000502) calcium ion transport				

Pattern in *S. x townsendii*	additive	Pattern in *S. x neyrautii*	transgressive up-regulated	Nb contigs: 1,333 (12.5%)
GO:0034637 (0.00335) cellular carbohydrate metabolic process				
GO:0009101 (1.84e-08) glycoprotein biosynthetic process				
GO:0006486 (1.84e-08) protein amino acid glycosylation				
GO:0006096 (0.000238) glycolysis				
GO:0032787 (0.000577) monocarboxylic acid metabolic process				
GO:0046686 (8.43e-09) response to cadmium ion				
GO:0009651 (0.00179) response to salt stress				
GO:0007242 (0.00784) intracellular signaling cascade				
GO:0009630 (1.88e-05) gravitropism				
GO:0006886 (8.77e-07) intracellular protein transport				
GO:0048513 (0.000465) organ development				
GO:0048229 (0.00901) gametophyte development				
GO:0009791 (0.000348) post-embryonic development				
GO:0010154 (0.00561) fruit development				
GO:0006396 (0.000712) RNA processing				
GO:0006468 (0.000838) protein amino acid phosphorylation				
GO:0007033 (0.000707) vacuole organization				
GO:0007030 (0.000162) Golgi organization				

Supplementary Table 8. Biological functions (list of enriched GO-terms) of differentially expressed genes between *S. x townsendii* and *S. anglica*. GO-terms are significantly enriched when adjusted p-value (in brackets) is lower than 0.01. Number of contigs corresponds to the number of genes DE between species.

S. anglica > S. x townsendii	**Nb contigs: 7,230**
Epidermis development	GO:0048469 (0.000956) cell maturation<br>GO:0008544 (3.7e-07) epidermis development<br>GO:0048764 (3.85e-06) trichoblast maturation<br>GO:0010090 (8.36e-08) trichome morphogenesis<br>GO:0048765 (3.85e-06) root hair cell differentiation
Gravitropism	GO:0009630 (9.62e-13) gravitropism
Fatty acid oxidation	GO:0006635 (6.36e-07) fatty acid beta-oxidation<br>GO:0006631 (1.16e-07) fatty acid metabolic process<br>GO:0009062 (0.000247) fatty acid catabolic process
Glycolysis process	GO:0006094 (4.68e-12) gluconeogenesis<br>GO:0006007 (3.91e-13) glucose catabolic process<br>GO:0006096 (8.21e-16) glycolysis
Protein modification process	GO:0006498 (0.009) N-terminal protein lipidation<br>GO:0006499 (0.009) N-terminal protein myristoylation<br>GO:0006511 (1.8e-09) ubiquitin-dependent protein catabolic process<br>GO:0016567 (0.00208) protein ubiquitination<br>GO:0016926 (0.00183) protein desumoylation<br>GO:0046777 (0.000231) protein amino acid autophosphorylation
Negative regulation / Gene silencing	GO:0035196 (0.0052) production of miRNAs involved in gene silencing by miRNA<br>GO:0016458 (2.12e-05) gene silencing<br>GO:0045892 (1.31e-06) negative regulation of transcription, DNA-dependent<br>GO:0006342 (3.24e-07) chromatin silencing<br>GO:0016481 (1.31e-06) negative regulation of transcription<br>GO:0045814 (3.16e-07) negative regulation of gene expression, epigenetic
Protein transport	GO:0006888 (0.000158) ER to Golgi vesicle-mediated transport<br>GO:0016558 (0.00931) protein import into peroxisome matrix<br>GO:0006623 (3.06e-06) protein targeting to vacuole<br>GO:0048193 (2.25e-16) Golgi vesicle transport
Calcium transport	GO:0070838 (7.17e-05) divalent metal ion transport<br>GO:0006816 (1.46e-05) calcium ion transport
Proteasome assembly	GO:0043248 (4.86e-05) proteasome assembly<br>GO:0080129 (0.000225) proteasome core complex assembly
Response to cadmium	GO:0010038 (1.35e-21) response to metal ion<br>GO:0046686 (1.89e-25) response to cadmium ion
Response and hydrogen peroxide process	GO:0042542 (2.73e-05) response to hydrogen peroxide<br>GO:0000302 (1.3e-06) response to reactive oxygen species<br>GO:0042743 (2.23e-07) hydrogen peroxide metabolic process<br>GO:0050665 (0.000259) hydrogen peroxide biosynthetic process
Response to salt stress	GO:0006972 (9.66e-05) hyperosmotic response<br>GO:0009651 (1.23e-15) response to salt stress
Response to hormone stimulus	GO:0009719 (5.19e-05) response to endogenous stimulus<br>GO:0009725 (0.00017) response to hormone stimulus<br>GO:0009737 (5.94e-05) response to abscisic acid stimulus<br>GO:0071365 (0.00385) cellular response to auxin stimulus<br>GO:0010033 (6.82e-15) response to organic substance
Response to other organism	GO:0051707 (8.11e-12) response to other organism<br>GO:0042742 (0.00461) defense response to bacterium<br>GO:0009620 (0.0041) response to fungus

S. anglica < S. x townsendii	**Nb contigs: 6,989**
Chloroplast development	GO:0045036 (0.00965) protein targeting to chloroplast<br>GO:0010027 (3.61e-07) thylakoid membrane organization<br>GO:0009658 (7.35e-06) chloroplast organization<br>GO:0015995 (0.00259) chlorophyll biosynthetic process
Sulfur biosynthetic process	GO:0044272 (3.13e-06) sulfur compound biosynthetic process<br>GO:0000096 (0.00616) sulfur amino acid metabolic process
Starch metabolic process	GO:0009250 (1.34e-06) glucan biosynthetic process<br>GO:0019252 (0.00122) starch biosynthetic process
Glycolysis process	GO:0006094 (3.17e-07) gluconeogenesis<br>GO:0006007 (9.87e-15) glucose catabolic process<br>GO:0006096 (9.46e-08) glycolysis
Response to stress	GO:0006950 (4.69e-26) response to stress<br>GO:0009414 (0.00127) response to water deprivation<br>GO:0051707 (8.11e-12) response to other organism<br>GO:0042742 (0.000495) defense response to bacterium<br>GO:0009620 (0.0012) response to fungus
Response to light stimulus	GO:0009416 (6.34e-08) response to light stimulus<br>GO:0010218 (0.00989) response to far red light
NADP metabolic process	GO:0046496 (3.58e-06) nicotinamide nucleotide metabolic process<br>GO:0006739 (2.2e-06) NADP metabolic process<br>GO:0006098 (5.61e-06) pentose-phosphate shunt

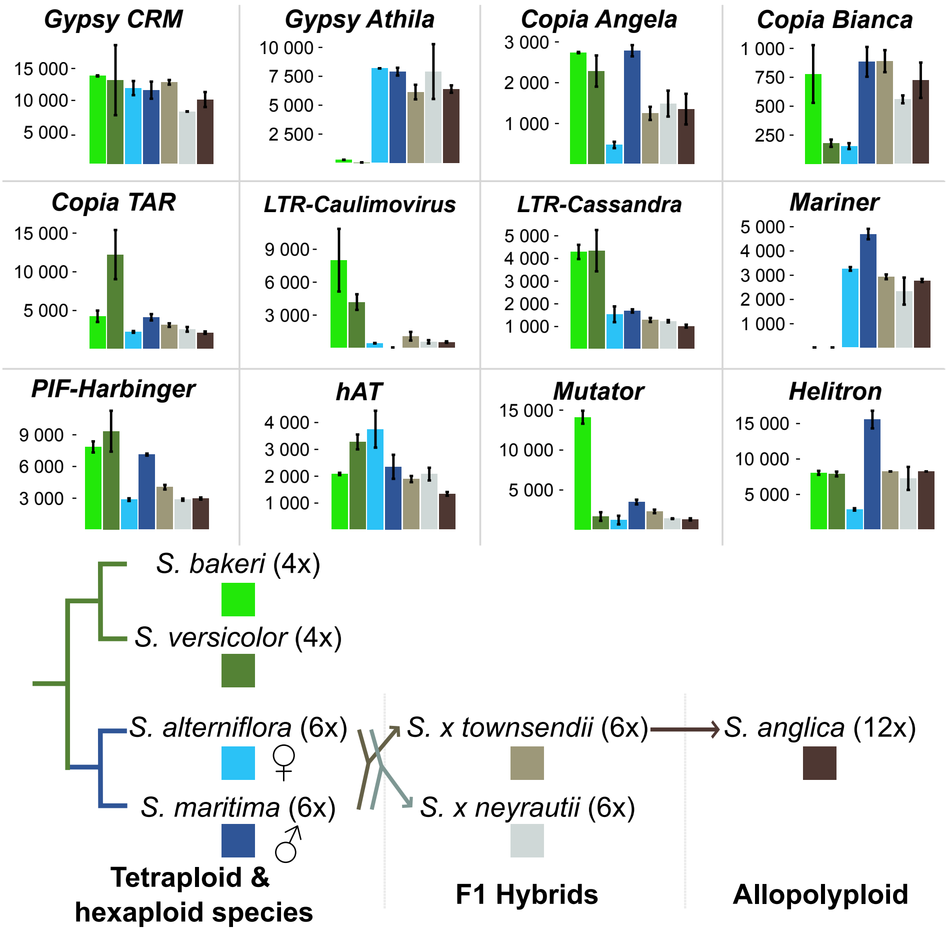

Supplementary Figure 4. Expression of lowest expressed transposable elements in *Spartina* genome.
